# Supplementary material for: Ventral pallidum GABA and glutamate neurons drive approach and avoidance through distinct modulation of VTA cell types
Source: Nat Commun. 2024 May 18;15:4233. doi: 10.1038/s41467-024-48340-y (PMC11102457; doi:10.1038/s41467-024-48340-y)
Supplement: Supplementary file 1 — Supplementary Information [file 41467_2024_48340_MOESM1_ESM.pdf]

Supplementary Materials for

Ventral pallidum GABA and glutamate neurons drive approach and avoidance through distinct modulation of VTA cell types

Lauren Faget *et al.*

*Corresponding author. Email: thnasko@health.ucsd.edu

This PDF file includes:

Figs. S1 to S10
Tables S1 to S2

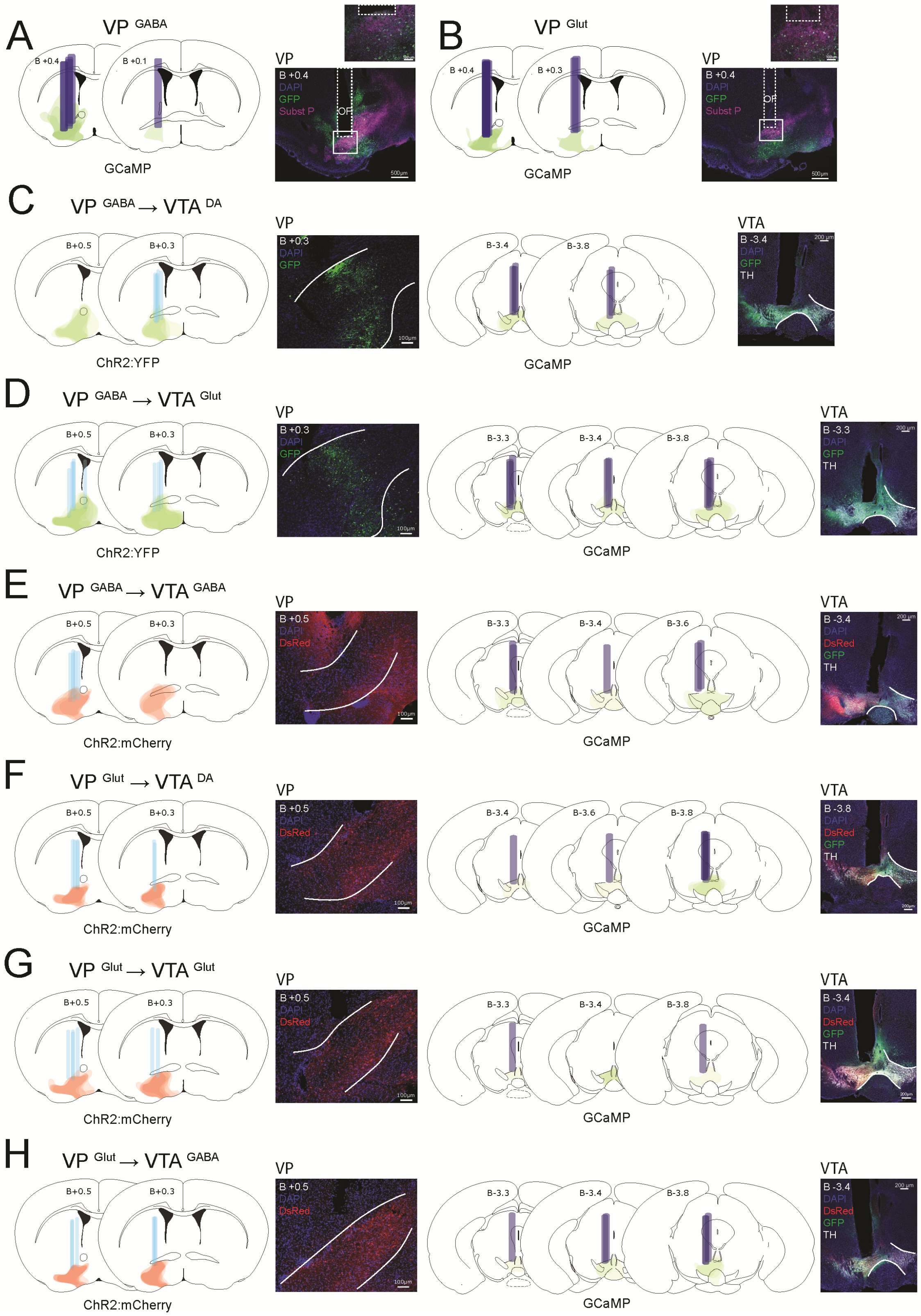

A
VP GABA
B +0.4
B +0.1
GCaMP
VP
B +0.4
DAPI
GFP
Subst P
OF
500µm
B
VP Glut
B +0.4
B +0.3
GCaMP
VP
B +0.4
DAPI
GFP
Subst P
OF
500µm
C
VP GABA → VTA DA
B+0.5
B+0.3
ChR2:YFP
VP
B +0.3
DAPI
GFP
100µm
B-3.4
B-3.8
GCaMP
VTA
B -3.4
DAPI
GFP
TH
200 µm
D
VP GABA → VTA Glut
B+0.5
B+0.3
ChR2:YFP
VP
B +0.3
DAPI
GFP
100µm
B-3.3
B-3.4
B-3.8
GCaMP
VTA
B -3.3
DAPI
GFP
TH
200 µm
E
VP GABA → VTA GABA
B+0.5
B+0.3
ChR2:mCherry
VP
B +0.5
DAPI
DsRed
100µm
B-3.3
B-3.4
B-3.6
GCaMP
VTA
B -3.4
DAPI
DsRed
GFP
TH
200 µm
F
VP Glut → VTA DA
B+0.5
B+0.3
ChR2:mCherry
VP
B +0.5
DAPI
DsRed
100µm
B-3.4
B-3.6
B-3.8
GCaMP
VTA
B -3.8
DAPI
DsRed
GFP
TH
200µm
G
VP Glut → VTA Glut
B+0.5
B+0.3
ChR2:mCherry
VP
B +0.5
DAPI
DsRed
100µm
B-3.3
B-3.4
B-3.8
GCaMP
VTA
B -3.4
DAPI
DsRed
GFP
TH
200µm
H
VP Glut → VTA GABA
B+0.5
B+0.3
ChR2:mCherry
VP
B +0.5
DAPI
DsRed
100µm
B-3.3
B-3.4
B-3.8
GCaMP
VTA
B -3.4
DAPI
DsRed
GFP
TH
200 µm

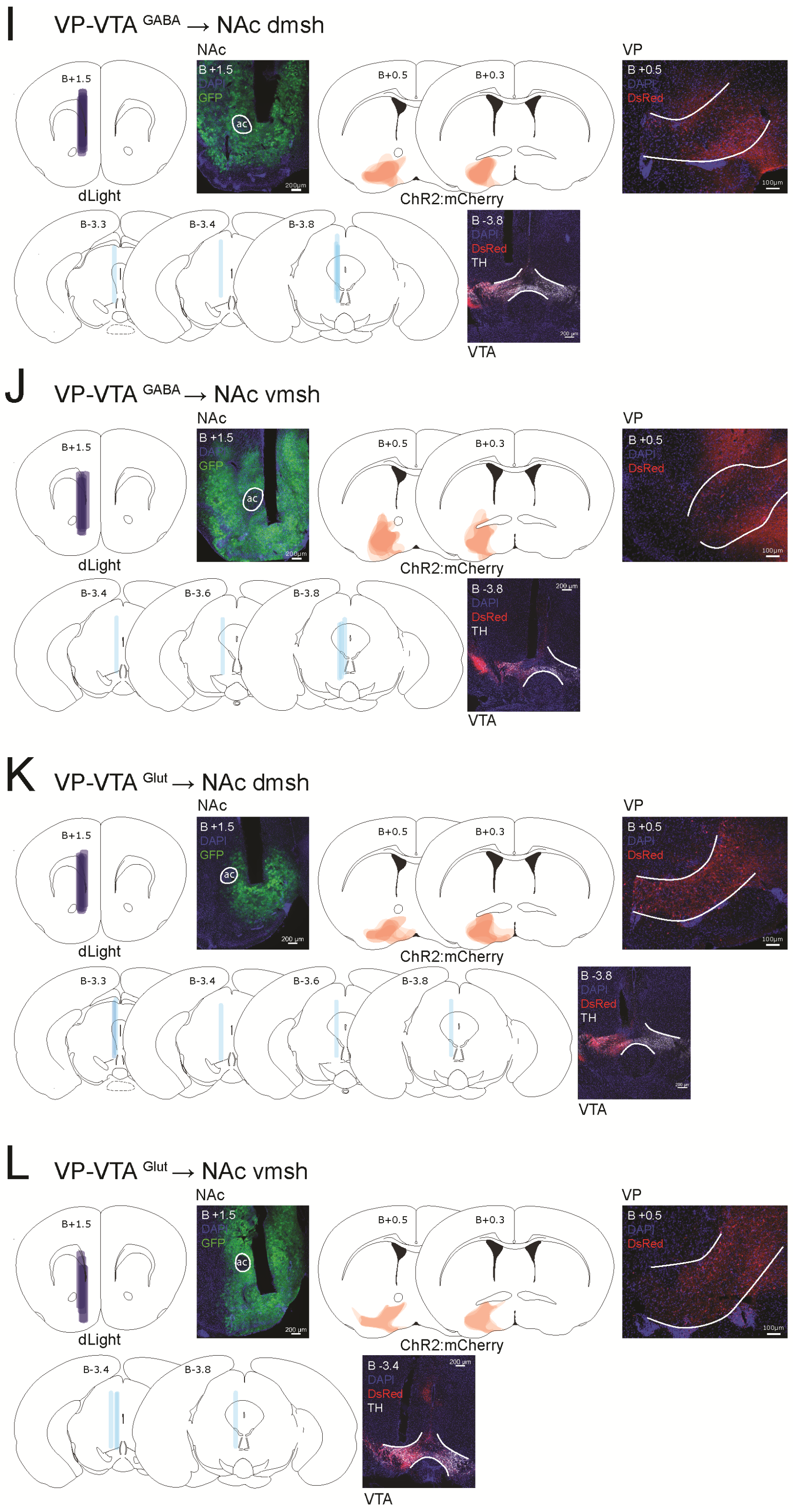

Fig. S1. Histological validation of reporter/opsin expression and fiber tract placements. A-L. Coronal sections showing example images of GCaMP reporter in VP or VTA, ChR2 opsin in VP or VTA, and/or dLight reporter in NAc for each experiment as indicated. GFP and DsRed

immunoreactivity amplified viral GFP/YFP and mCherry fluorescence respectively. Some images show substance P immunoreactivity used to demark VP borders, Tyrosine hydroxylase (TH) to demark VTA, and DAPI nuclear stain is shown in blue. Diagrams schematize spread of indicated opsin or reporter and optic fiber (OF) placements at various points relative to Bregma (B) in mm; ac, anterior commissure. See Table S1 for details on viral and genetic strategies. Related to Fig. 1 through 6.

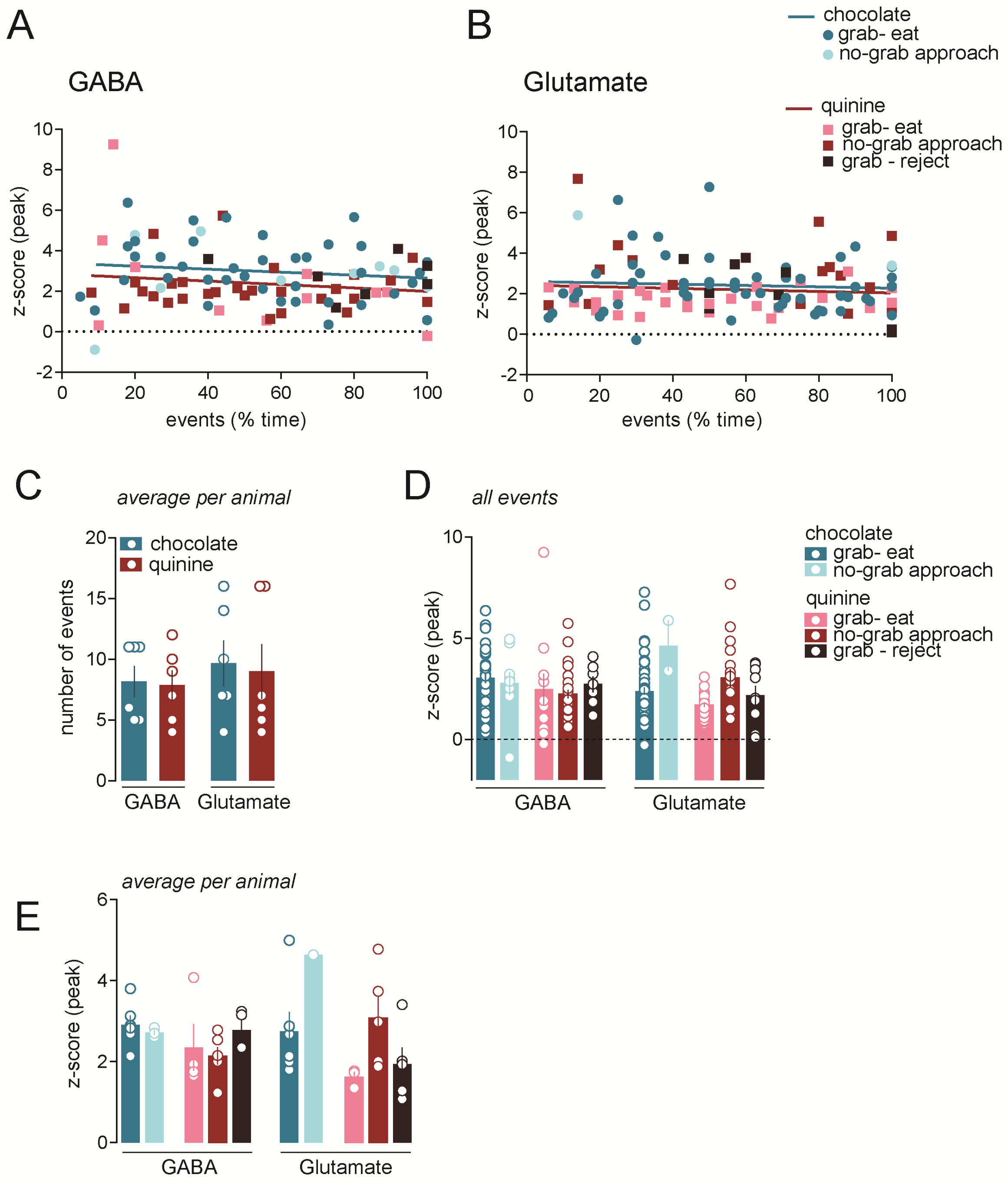

Fig. S2. VP cell type responses during interaction with positive- and negative-valence food. Peak GCaMP fluorescence recorded from **A**. VP GABA and **B**. VP Glutamate neurons during the first second of 'grab-eat', 'grab-reject', and 'no-grab approach' to chocolate-flavored and quinine-coated pellets. Individual data normalized to the number of events per subject across time (n=6 mice/group). **C**. Average +/- sem number of events per animal by pellet type and VP cell type (Two-way ANOVA, no effect of pellet type, $F(1,20)=0.1$, $p=0.8$; no effect of VP cell type, $F(1,20)=0.6$, $p=0.4$; no interaction, $F(1,20)=0.01$, $p=0.9$). **D**. Average +/- sem peak z-score per event by pellet type, event type and VP cell type. Two-way ANOVA, no effect of VP cell type,

$F(1,197)=0.3$, $p=0.6$; no effect of event type, $F(4,197)=2.2$, $p=0.07$; interaction, $F(4,197)=3.1$, $p=0.02$. **E**. Average +/- sem peak z-score per animal by pellet type, event type and VP cell type. Two-way ANOVA, no effect of VP cell type, $F(1,33)=0.6$, $p=0.5$; no effect of event type $F(4,33)=2.5$, $p=0.06$; interaction, $F(4,33)=2.7$, $p=0.049$. Related to Fig. 2. Source data are provided as a Source Data file.

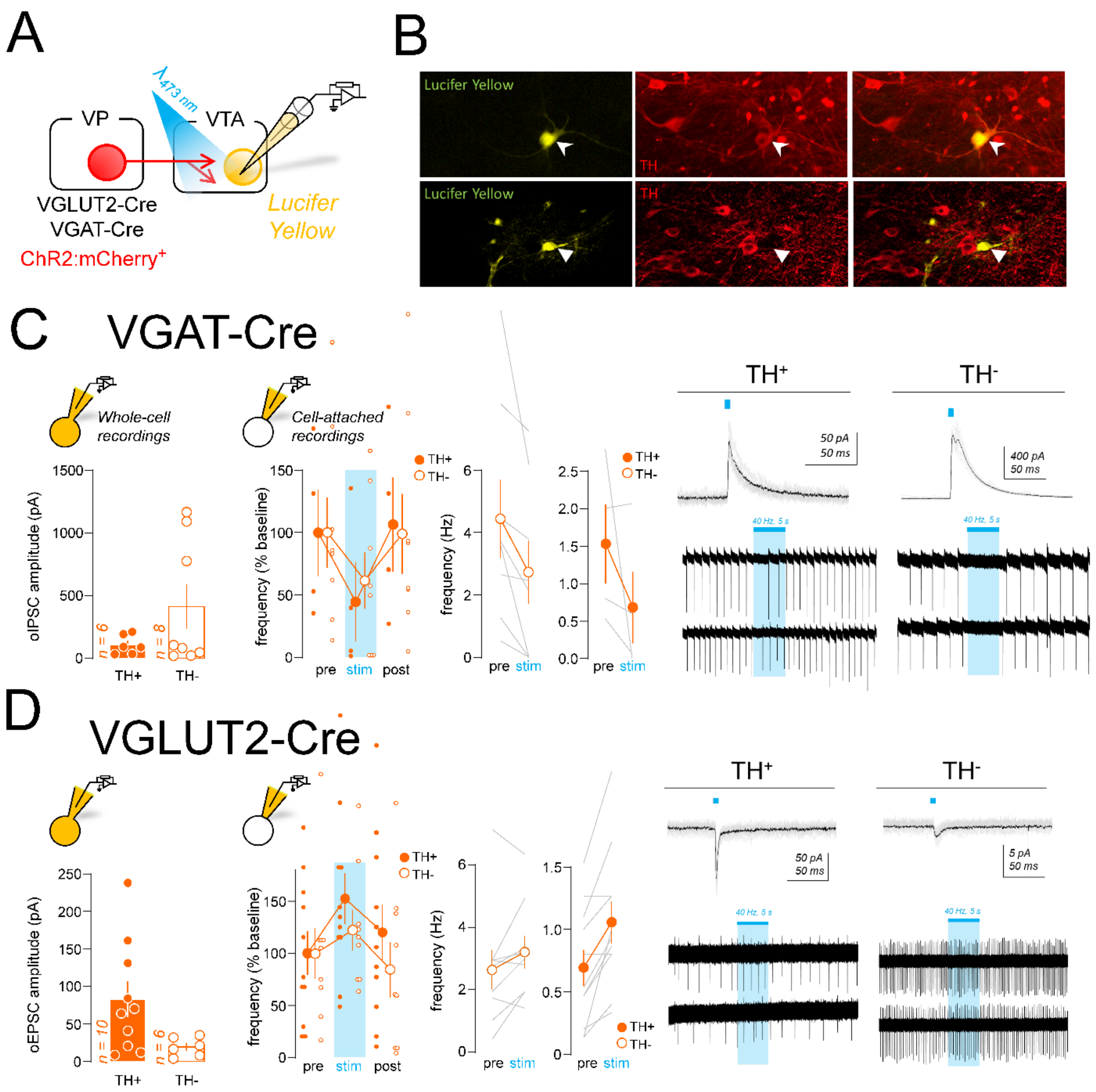

Fig. S3. Ex vivo recordings of VP cell type connectivity to VTA dopamine and non-dopamine neurons. A. Schematic showing experimental design with Cre-dependent expression of ChR2:mCherry in VP cell types and *ex vivo* slice recordings from VTA neurons filled with Lucifer Yellow. **B**. Sections from VTA with recorded neurons filled with Lucifer Yellow were post-hoc processed for IHC to stain for the dopamine marker TH. Top row shows an example TH+ (arrows) and bottom row shows example TH- (arrows) neuron. **C**. Electrophysiological recordings in slices from VGAT-Cre mice. Left: Whole cell recordings showing amplitude of photo-evoked IPSCs elicited on TH+ (n=6 cells) and TH- (n=8 cells) neurons (Two-tailed unpaired t-test, $t(12)=1.4$; $p=0.2$). Right: Cell attached recordings showing percentage change in firing rate from a 5-s baseline (pre-stimulation period), during optostimulation (5 s, 40 Hz) and 5-s post-stimulation recovery period for TH+ (n=4 cells) and TH- (n=8 cells) VTA neurons (Two-way ANOVA, effect of stimulation, $F(2,20)=6.5$, $p=0.007$; no effect of VTA cell type, $F(1,10)= 0.005$, $p=0.9$; no

interaction $F(2,20)=0.3$, $p=0.7$). Effect of 40-Hz photostimulation on firing rates (in Hz) on TH+ and TH- VTA neurons is also shown. Far right inset shows example traces of optogenetic-triggered IPSCs in TH+ and TH- VTA neurons as well as representative traces showing the effect of 40 Hz photostimulation on TH+ and TH- neuron firing. **D**. Electrophysiological recordings in slices from VGLUT2-Cre mice. Left: Amplitude of photo-evoked EPSCs elicited on TH+ (n=10 cells) and TH- (n=6 cells) neurons (Two-tailed unpaired t-test, $t(14)=2.1$; $p=0.06$). Right: Cell attached recordings showing percentage change in firing rate from 5-s baseline, during optostimulation (5 s, 40 Hz), and 5-s post-stimulation recovery for TH+ (n=11 cells) and TH- (n=9 cells) VTA neurons (Two-way ANOVA, effect of stimulation, $F(2,36)=11.5$, $p=0.0001$; no effect of VTA cell type $F(1,18)=0.4$, $p=0.5$; no interaction, $F(2,36)= 20.7$, $p=0.1$). Effect of optogenetic stimulation on firing rates (Hz) on TH+ and TH- VTA neurons is also shown. Far right displays representative traces of whole cell and cell-attached recordings. Data are shown as mean ± SEM. Related to Fig. 3 through 5. Source data are provided as a Source Data file.

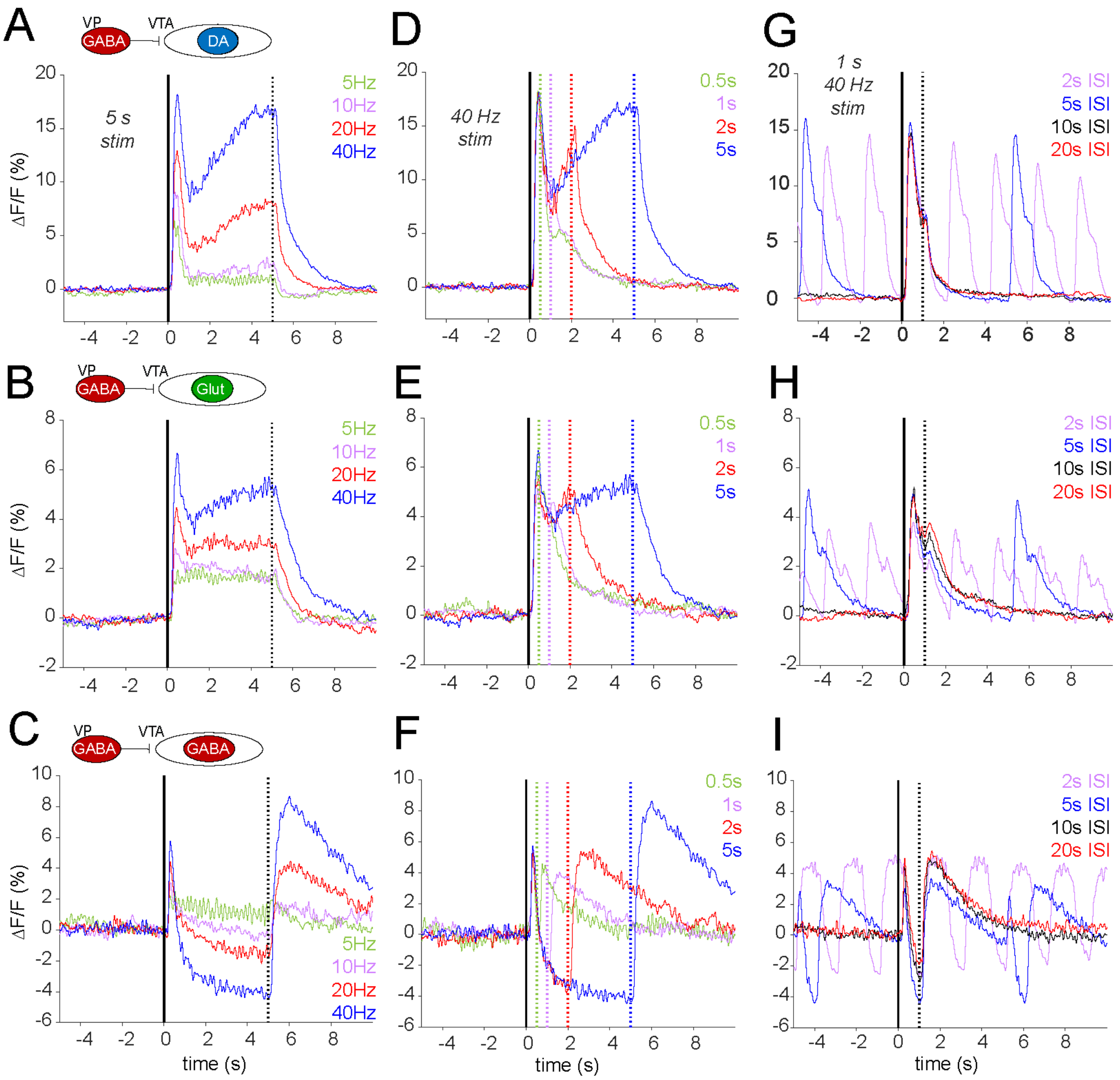

Fig. S4. VTA cell-type responses to passive optogenetic stimulation of VP GABA neurons at different durations, frequencies, and inter-stimulus intervals. Responses (percent of ΔF/F) of GCaMP-expressing **A.** VTA DA (n=5 mice), **B.** Glut (n=8 mice), and **C.** GABA (n=5 mice) neurons to different frequencies (5, 10, 20, and 40Hz) of VP GABA neuron stimulation (t=0) delivered for 5 s with a 20s inter-stimulation interval (ISI). **D.** VTA DA, **E.** Glut, and **F.** GABA neuron responses to 40 Hz stimulation for different durations (0.5, 1, 2, and 5s; onset at t=0s), with the end of each stimulation noted by a vertical dotted line of the corresponding color and 20s ISI. **G.** VTA DA, **H.** Glut, and **I.** GABA neuron responses to 40-Hz 1-s stimulation with variable ISI (2, 5, 10, and 20s). Note that with shorter ISIs signals have not returned to baseline prior to stimulation onset. Data are presented as mean (10 trials per condition, per animal). Related to Fig. 3 and 4.

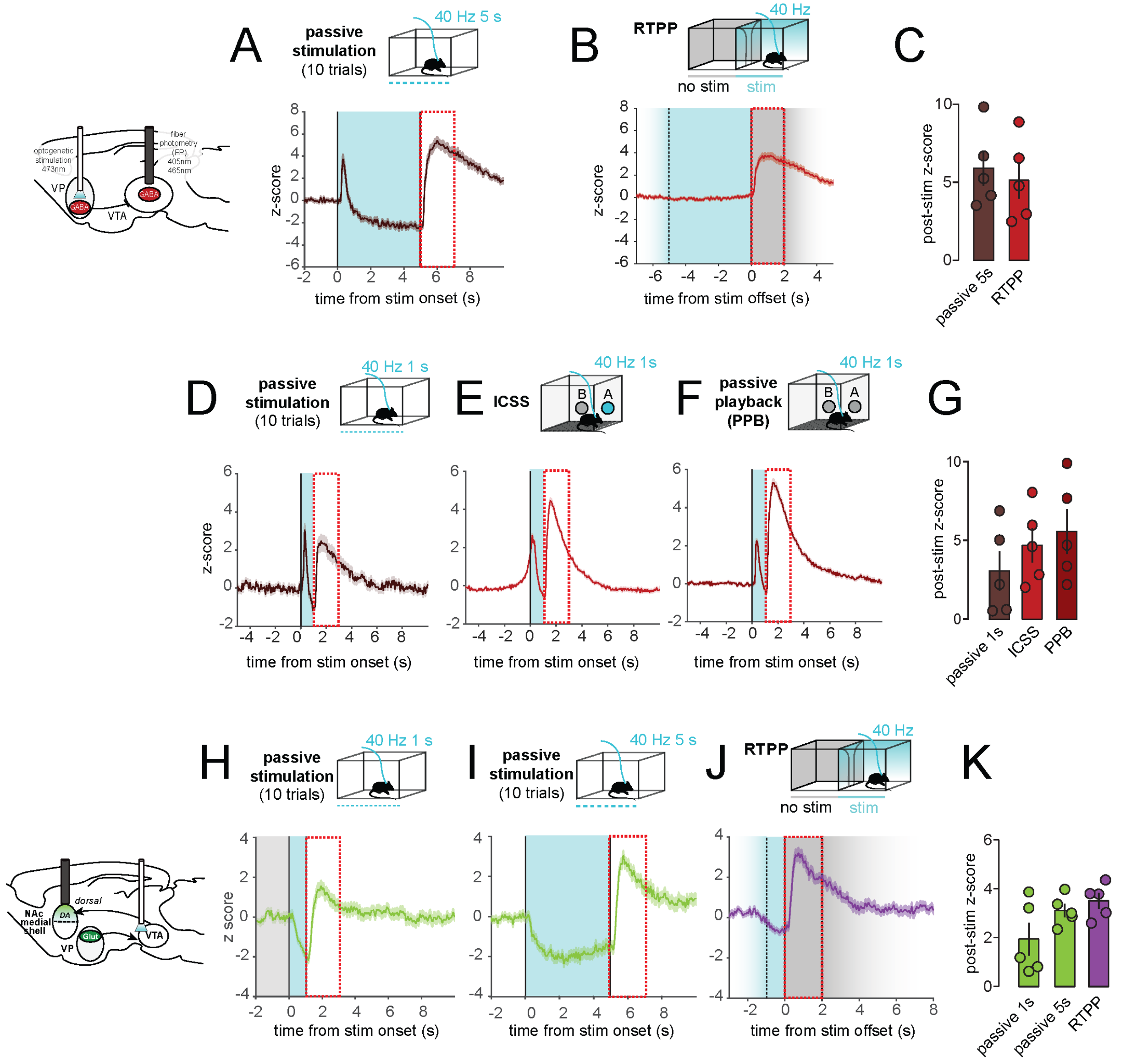

Fig. S5. Quantification of overshoot following optogenetic evoked inhibition of VTA GABA neurons or dopamine release. A-C. VTA GABA neuron peak fluorescence (z-score, GCaMP) during the 2s (dashed red box) following termination of VP GABA neuron stimulation (n=5 mice): 5 s passive stimulation or during RTPP on trials where mice spent a minimum of 5s in the active side followed by a minimum of 2s in the inactive side. Two-tailed paired t-test t(4)=0.9, p=0.4. **D-G**. VTA GABA neuron peak fluorescence (z-score, GCaMP) during the 2s (dashed red box) following termination of VP GABA neuron stimulation: 1s-passive stimulation, 1s self-stimulation during ICSS, or 1s stimulation during PPB. RM one-way ANOVA F(1,4)=1.7, p=0.3. **H-K**. Peak fluorescence of dopamine signal (z-score, dLight) in NAc dmsh during the 2s (dashed red box) following termination of VP Glut neuron stimulation (n=5 mice): 1s-passive stimulation, 5s-passive stimulation, or during RTPP on trials where mice spent a minimum of 1s in the active side followed by a minimum of 2s in the inactive side. RM one-way ANOVA F(1,4)=2.7, p=0.2. Traces and histograms are average ± SEM. Related to Fig. 3, 4 and 6. Source data are provided as a Source Data file.

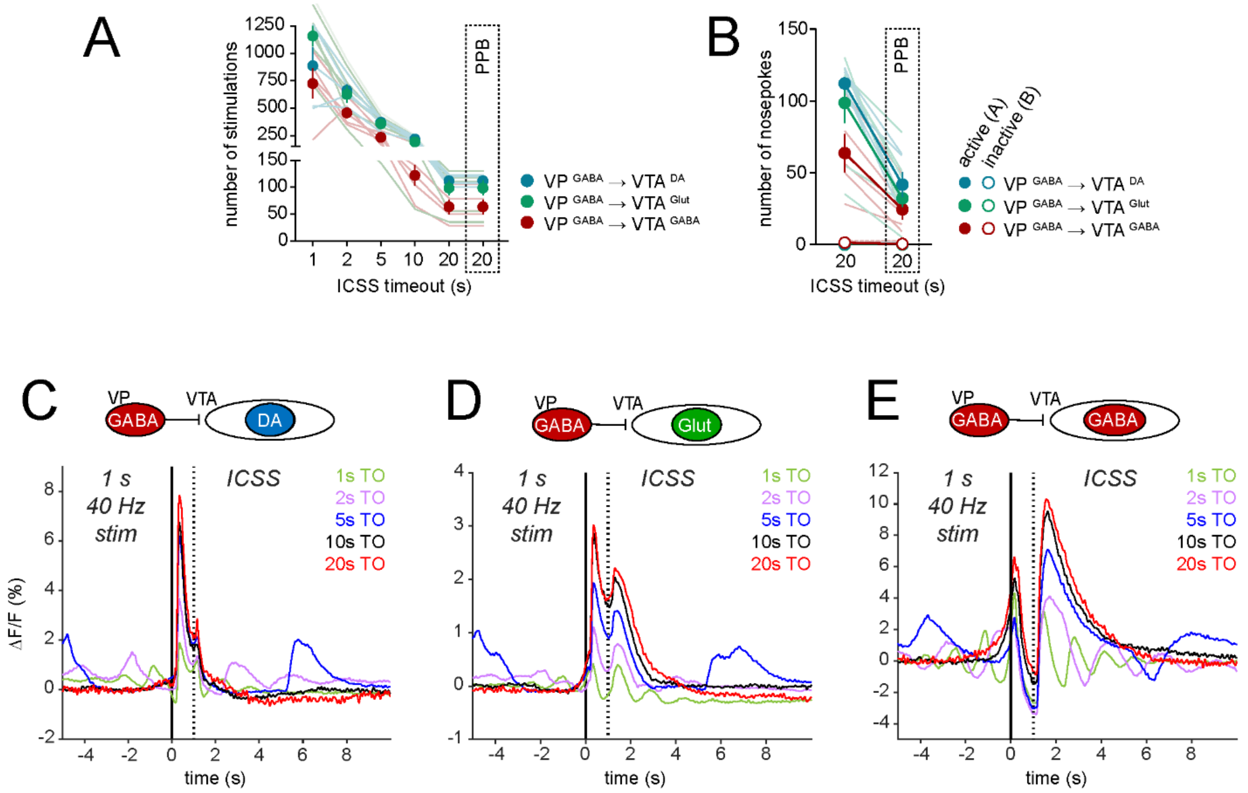

Fig. S6. VTA cell-type responses during VP GABA neuron ICSS with different timeout periods. A. Number of self-administered VP GABA neuron stimulations for each cohort of GCAMP recordings. An increasing timeout (TO) period was imposed between stimulation availability across daily sessions. The number of stimulations delivered during PPB is a replay of and thus identical to the 20s TO condition. Two-way ANOVA, effect of TO, $F(5,75)=127$, $p<0.0001$, effect of group, $F(2,15)=4.5$, $p=0.03$; interaction, $F(10,75)=3.4$, $p=0.001$. **B.** Number of nosepokes made into the active and inactive holes during the 20-s TO ICSS session and subsequent PPB. Three-way ANOVA; effect of session, $F(1,30)=46.7$, $p<0.0001$, effect of hole type, $F(1,30)=219.3$, $p<0.0001$, effect of group, $F(2,30)=4.7$, $p=0.02$**. C.** VTA DA (n=5 mice), **D.** Glut (n=8 mice) and **E.** GABA (n=5 mice) neuron GCaMP responses (percent of ΔF/F) to ICSS (t=0s) during the sessions with different TO periods imposed. Note that due to the high rate of ICSS, when TO periods were short GCaMP signals had not returned to baseline prior to stimulation onset. Data are presented as A & B, mean ± SEM; C-E, mean. Related to Fig. 4. Source data are provided as a Source Data file.

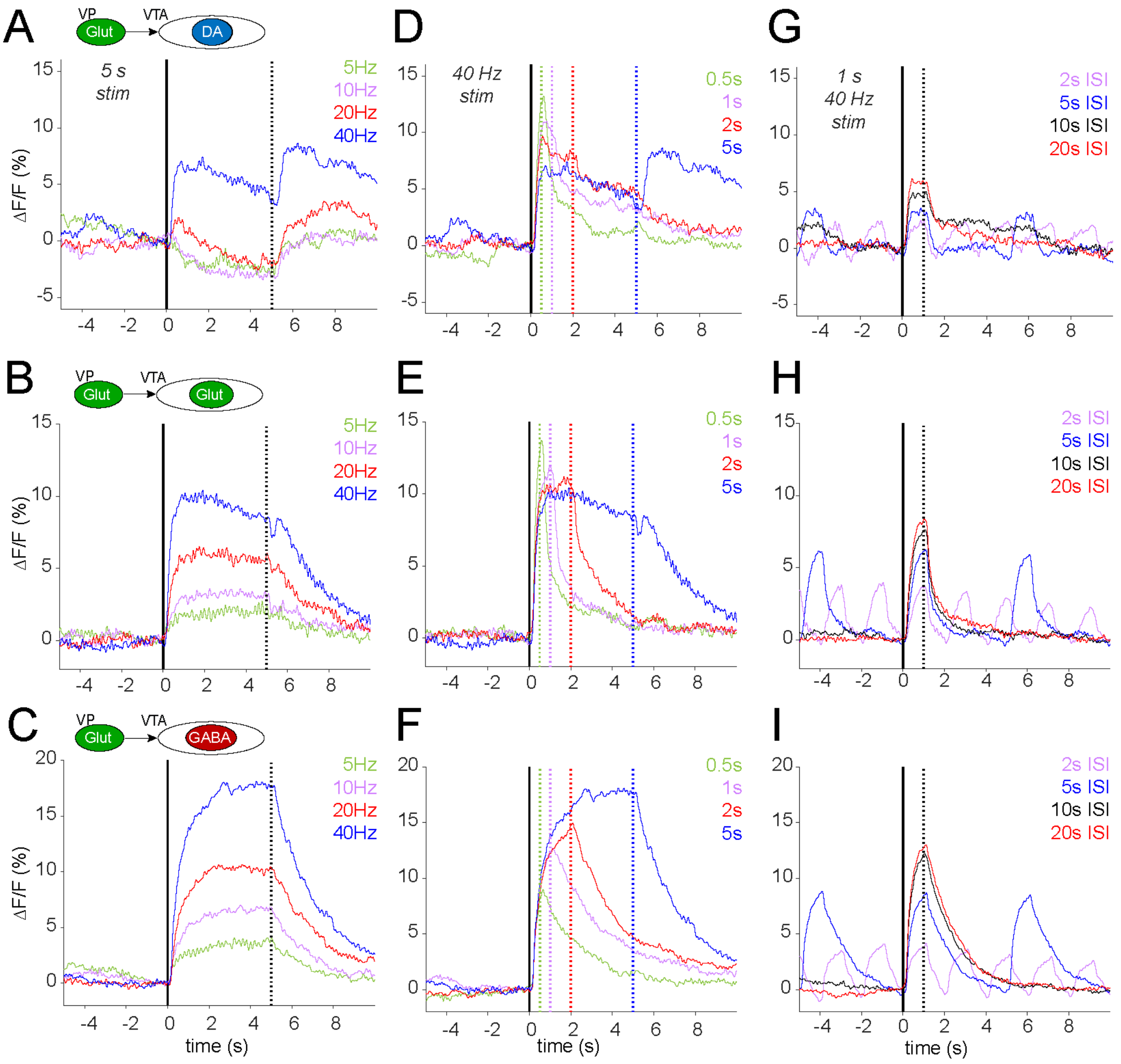

Fig. S7. VTA cell-type responses to passive optogenetic stimulation of VP Glut neurons at different durations, frequencies and inter-stimulus intervals. Responses (percent of ΔF/F) of GCaMP-expressing **A**. VTA DA (n=6 mice), **B.** Glut (n=7 mice), and **C.** GABA (n=6 mice) neurons to different frequencies (5, 10, 20, and 40Hz) of VP Glut neuron stimulation (t=0) delivered for 5 s with a 20s inter-stimulation interval (ISI). **D.** VTA DA, **E.** Glut, and **F.** GABA neuron responses to 40 Hz stimulation for different durations (0.5, 1, 2, and 5s; onset at t=0s), with the end of each stimulation noted by a vertical dotted line of the corresponding color, and 20s ISI. **G.** VTA DA, **H.** Glut, and **I.** GABA neuron responses to 40-Hz 1-s stimulation with variable ISI (2, 5, 10, and 20s). Note that with shorter ISIs signals have not returned to baseline prior to stimulation onset. Data are presented as mean (10 trials per condition, per animal). Related to Fig. 5.

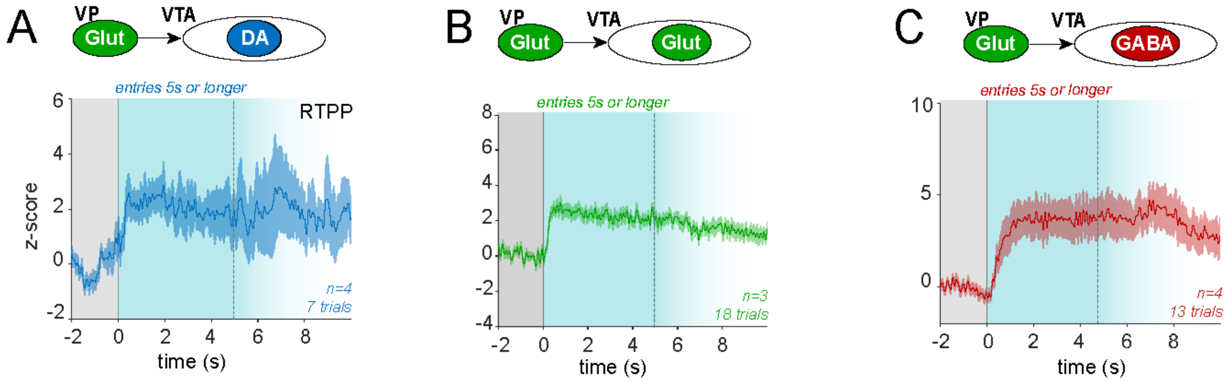

Fig. S8. VTA cell-type responses to more sustained VP Glut neuron stimulation during RTPP assay. In this experiment active side entry activates VP Glut neurons and because this is aversive mice make relatively few and relatively brief entries into the active side. However, some of the mice do make a few more sustained entries and these are the GCaMP responses (z-score) of **A.** VTA DA (n=4 mice; 7 trials), **B.** VTA Glut (n=3 mice; 18 trials), and **C.** VTA GABA (n=4 mice; 13 trials) neurons for entries sustained for $\geq$5s in the stimulation side (blue shading, fades after 5s) and preceded by $\geq$2s in the no- stimulation side (gray shading). Data are presented as mean ± SEM. Related to Fig. 5.

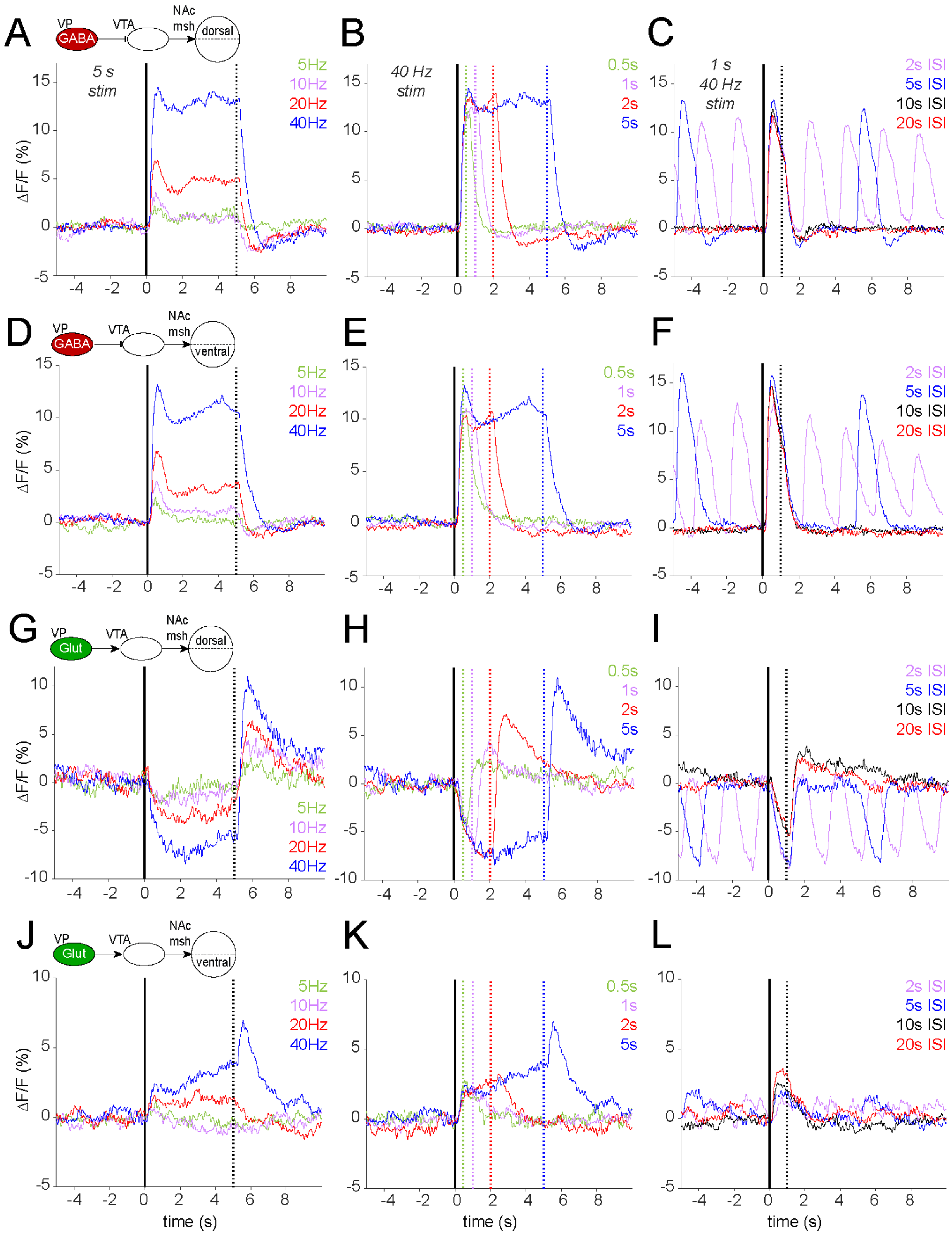

Fig. S9. Dopamine release in the dorsal and ventral medial shell of NAc subregions in response to varied durations, frequencies and inter-stimulation intervals of optogenetic

stimulation of VP GABA and glutamate neuron terminals in VTA. DA dLight responses (percent of ΔF/F) in dmsh of NAc to stimulation (onset t=0) of VP GABA terminals in VTA (n=6 mice) with varied **A.** frequency (fixed at 5s duration, 20s ISI), **B.** duration (fixed at 40Hz, 20s ISI), or **C.** ISI (fixed at 40Hz, 1s duration). DA dLight responses in vmsh of NAc to stimulation of VP GABA terminals in VTA (n=5 mice) with varied **D.** frequency (fixed at 5s duration, 20s ISI), **E.** duration (fixed at 40Hz, 20s ISI), or **F.** ISI (fixed at 40Hz, 1s duration). DA dLight responses in dmsh of NAc to stimulation of VP Glut terminals in VTA (n=5 mice) with varied **G.** frequency (fixed at 5s duration, 20s ISI), **H.** duration (fixed at 40Hz, 20s ISI), or **I.** ISI (fixed at 40Hz, 1s duration). DA dLight responses in vmsh of NAc to stimulation (onset t=0) of VP GABA terminal in VTA (n=4 mice) with varied **J.** frequency (fixed at 5s duration, 20s ISI), **K.** duration (fixed at 40Hz, 20s ISI), or **L.** ISI (fixed at 40Hz, 1s duration). Data are presented as mean (10 trials per condition, and per animal). Related to Fig. 6.

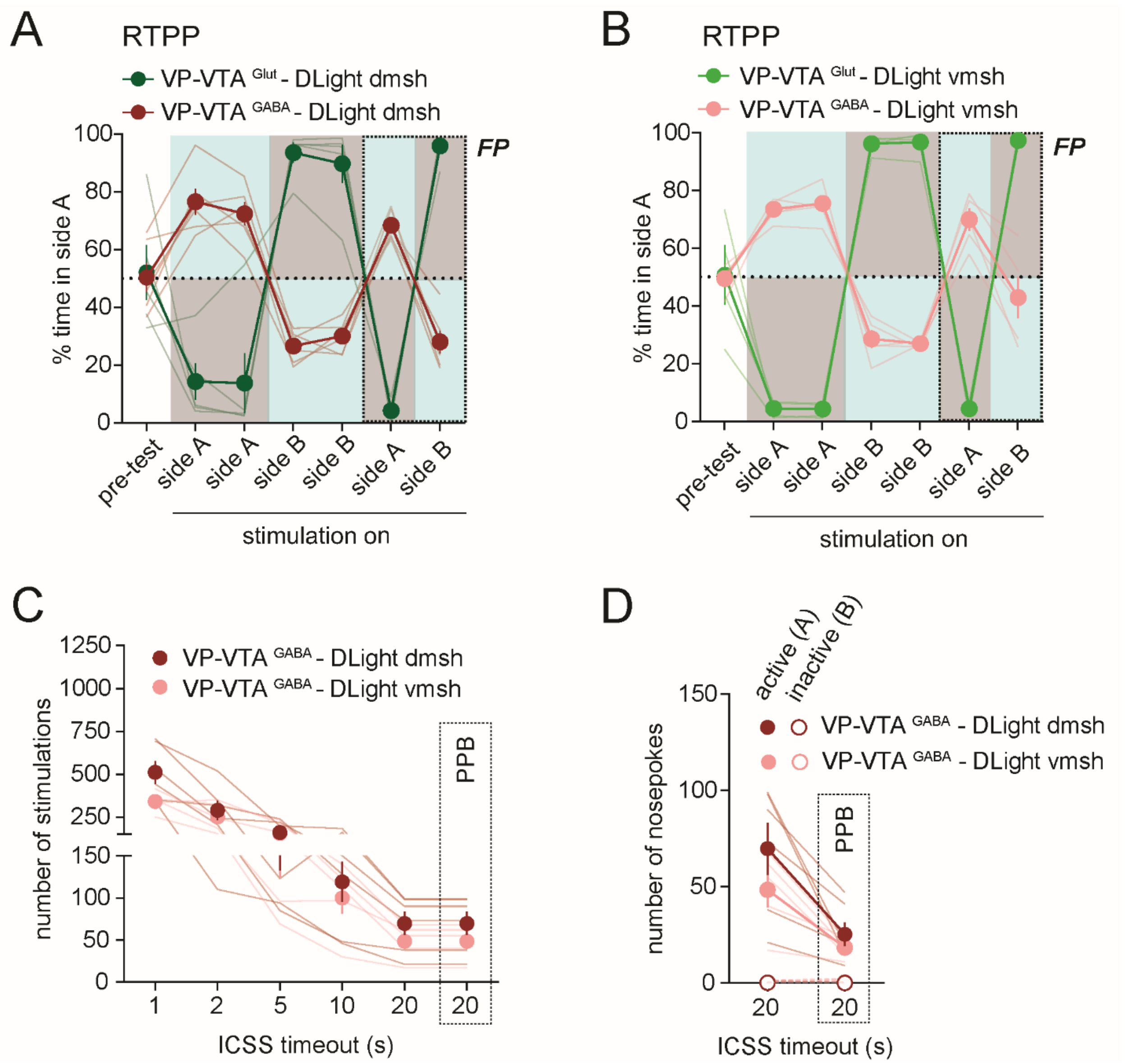

Fig. S10. Behavioral responses to stimulation of VP GABA and Glut terminals in VTA. Fraction of time spent in side A during RTPP for stimulation of VP Glut or GABA terminals in VTA, when recording DLight responses from **A**. NAc dmsh (Glut, n=5; GABA, n=6 mice) and **B.** NAc vmsh (Glut, n=4; GABA, n=5 mice). Blue shading represents stimulated side; recordings were made only during the last two sessions (annotated FP). NAc dmsh (A), two-way ANOVA, effect of session, $F(6,54)=7.5$, $p<0.0001$; no effect of VP cell type, $F(1,9)=0.5$, $p=0.5$; interaction, $F(6,54)=76.4$, $p<0.0001$. NAc vmsh (B), two-way ANOVA, effect of session, $F(6,42)= 24.0$, $p<0.0001$; no effect of VP cell type, $F(1,7)=0.7$, $p=0.4$; interaction, $F(6,42)=148.5$, $p<0.0001$. **C**. Number of nosepokes leading to self-stimulation of VP GABA terminals in VTA for each cohort of mice used in DLight recordings and for each timeout (TO) period tested (1, 2, 5, 10, and 20s). The number of stimulations delivered during passive playback (PPB) is a replay of and thus identical to the 20s TO condition. Two-way ANOVA, effect of TO, $F(5,45)=65$, $p<0.0001$; no effect of group, $F(1,9)=1.6$, $p=0.2$; interaction, $F(5,45)=6.3$; $p=0.02$. **D**. Number of nosepokes made into the active and inactive holes during the 20-s TO ICSS session and subsequent PPB

session. Three-way ANOVA; effect of session, $F(1,9)=22.8$, $P=0.001$; effect of hole type, $F(1,9)=57.3$, $p<0.0001$; no effect of group, $F(1,9)=1.7$, $p=0.2$. Data are presented as mean ± SEM. Related to Fig. 6. Source data are provided as a Source Data file.

Table S1. Combinatorial Cre- or FLP-dependent adeno-associated virus (AAV) vectors strategies to achieve expression of ChR2 in VP cell types, GCaMP in VTA cell types, and ubiquitous expression of dLight in NAc. Related to Fig. 1 to 7.

Cell type / Pathway	Genotype	n=	Virus in VP	Virus in VTA	Virus in NAc
**VP GABA**	VGAT Cre/Cre	6 (5m, 1f)	AAV5-DIO-GCaMP6f		
**VP Glut**	VGLUT2 Cre/Cre	7 (4m, 3f)	AAV5-DIO-GCaMP6f		
**VP GABA → VTA DA**	VGAT +/FlpO ; DAT +/Cre	5 (2m, 3f)	AAVDJ-fDIO-ChR2:YFP	AAV5-DIO-GCaMP6f	
**VP GABA → VTA Glut**	VGAT +/FlpO ; VGLUT2 +/Cre	8 (3m, 5f)	AAVDJ-fDIO-ChR2:YFP	AAV5-DIO-GCaMP6f	
**VP GABA → VTA GABA**	VGAT Cre/Cre	5 (2m, 3f)	AAV5-DIO-ChR2:mCherry	AAV5-DIO-GCaMP6f	
**VP Glut → VTA DA**	VGLUT2 +/Cre ; DAT +/FlpO	6 (3m, 3f)	AAV5-DIO-ChR2:mCherry	AAVDJ-fDIO-GCaMP7f	
**VP Glut → VTA Glut**	VGLUT2 Cre/Cre	7 (3m, 4f)	AAV5-DIO-ChR2:mCherry	AAV5-DIO-GCaMP6f	
**VP Glut → VTA GABA**	VGLUT2 +/Cre ; VGAT +/FlpO	6 (3m, 3f)	AAV5-DIO-ChR2:mCherry	AAVDJ-fDIO-GCaMP7f	
**VP-VTA GABA → NAc dmsh**	VGAT Cre/Cre	6 (3m, 3f)	AAV5-DIO-ChR2:mCherry		AAV5-CAG-dLight1.1
**VP-VTA GABA → NAc vmsh**	VGAT Cre/Cre	5 (2m, 3f)	AAV5-DIO-ChR2:mCherry		AAV5-CAG-dLight1.1
**VP-VTA Glut → NAc dmsh**	VGLUT2 Cre/Cre	5 (4m, 1f)	AAV5-DIO-ChR2:mCherry		AAV5-CAG-dLight1.1
**VP-VTA Glut → NAc vmsh**	VGLUT2 Cre/Cre	4 (2m, 2f)	AAV5-DIO-ChR2:mCherry		AAV5-CAG-dLight1.1

Table S2. Statistics table. Related to Fig. 1 to 6. Source data are provided as Source Data files.

						variables				Statistical analysis					Post-hoc analysis			
**Fig**	**Panel**	**Test**	**VP cell type**	**VTA cell type**	**n= (m,f)**	**Independent**	**dependent**	**mean**	**sem**	**Type**	**factors**	**F or t value**	**p value**	**asterisks**	**Type**	**Comparison**	**p values**	**asterisks**
**1**	**A**	HFHS interaction	GABA		6	first 10 interactions<br>-<br>z-score peak	Baseline<br>Pre-event<br>Event<br>(time period)	-0.01<br>1.96<br>2.15	0.24<br>0.30<br>0.46	RM 1-way ANOVA	time period	F(2,5) = 19.1	0.005	**	Tukey	baseline vs. pre-event<br>baseline vs. event<br>pre-event vs. event	0.009<br>0.02<br>0.5	**<br>*<br>ns
					(5m)		Baseline<br>Pre-event<br>Event	0.07<br>1.92<br>2.17	0.27<br>0.36<br>0.56									
					(1f)		Baseline<br>Pre-event<br>Event	-0.42<br>2.19<br>2.21										
					6	last 10 interactions<br>-<br>z-score peak	Baseline<br>Pre-event<br>Event	-0.14<br>1.99<br>2.11	0.3<br>0.30<br>0.32	RM 1-way ANOVA	time period	F(2,5)=36.7	0.001	**	Tukey	baseline vs. pre-event<br>baseline vs. event<br>pre-event vs. event	0.006<br>0.002<br>0.4	**<br>**<br>ns
					(5m)		Baseline<br>Pre-event<br>Event	-0.04<br>2.01<br>2.19	0.34<br>0.37<br>0.38									
					(1f)		Baseline<br>Pre-event<br>Event	-0.65<br>1.88<br>1.71										
					6	z-score peak	First 10<br>Last 10	2.17<br>2.11	0.46<br>0.32	two-tailed paired *t-test*		t(5)= 0.1	0.9	ns				
					(5m)		First 10<br>Last 10	2.17<br>2.19	0.56<br>0.38									
					(1f)		First 10<br>Last 10	2.21<br>1.71										
	**B**	HFHS interaction	Glut		7	first 10 interactions<br>-<br>z-score peak	Baseline<br>Pre-event<br>Event	-0.42<br>1.99<br>1.86	0.21<br>0.26<br>0.21	RM 1-way ANOVA	time period	F(2,6)=41.7	< 0.0001	****	Tukey	baseline vs. pre-event<br>baseline vs. event<br>pre-event vs. event	0.001<br>0.001<br>0.7	**<br>**<br>ns
					(4m)		Baseline<br>Pre-event<br>Event	-0.55<br>2.33<br>2.16	0.12<br>0.25<br>0.08									
					(3f)		Baseline<br>Pre-event<br>Event	-0.23<br>1.55<br>1.45	0.49<br>0.4<br>0.41									
					7	last 10 interactions<br>-<br>z-score peak	Baseline<br>Pre-event<br>Event	-0.47<br>1.82<br>1.73	0.16<br>0.21<br>0.19	RM 1-way ANOVA	time period	F(2,6)=50.3	< 0.0001	****	Tukey	baseline vs. pre-event<br>baseline vs. event<br>pre-event vs. event	0.0006<br>0.0008<br>0.8	***<br>***<br>ns
					(4m)		Baseline<br>Pre-event<br>Event	-0.37<br>2.07<br>1.85	0.28<br>0.28<br>0.31									
					(3f)		Baseline<br>Pre-event<br>Event	-0.6<br>1.49<br>1.57	0.04<br>0.24<br>0.17									
					7	z-score peak	First 10<br>Last 10	1.86<br>1.73	0.21<br>0.19	two-tailed paired *t-test*		t(6)= 0.6	0.5	ns				
					(4m)		First 10<br>Last 10	2.16<br>1.85	0.08<br>0.31									

					(3f)		First 10<br>Last 10	1.45<br>1.57	0.41<br>0.17									
	**C**	Marble interacti on	GABA		6	first 10 interactions - z-score peak	Baseline<br>Pre-event<br>Event	0.21<br>2.19<br>2.47	0.22<br>0.34<br>0.35	RM 1-way ANOVA	time period	F(2,5)=27.5	0.002	**	Tukey	baseline vs. pre-event<br>baseline vs. event<br>pre-event vs. event	0.009<br>0.005<br>0.1	**<br>**<br>ns
					(5m)		Baseline<br>Pre-event<br>Event	0.15<br>2.33<br>2.65	0.26<br>0.38<br>0.36									
					(1f)		Baseline<br>Pre-event<br>Event	0.51<br>1.51<br>1.54										
					6	last 10 interactions - z-score peak	Baseline<br>Pre-event<br>Event	-0.02<br>1.06<br>1.07	0.3<br>0.15<br>0.15	RM 1-way ANOVA	time period	F(2,5)=11.2	0.02	*	Tukey	baseline vs. pre-event<br>baseline vs. event<br>pre-event vs. event	0.046<br>0.04<br>0.9	*<br>*<br>ns
					(5m)		Baseline<br>Pre-event<br>Event	0.13<br>1.09<br>1.06	0.31<br>0.18<br>0.18									
					(1f)		Baseline<br>Pre-event<br>Event	-0.78<br>0.93<br>1.11										
					6	z-score peak	First 10<br>Last 10	2.47<br>1.07	0.35<br>0.15	two-tailed paired *t-test*		t(5)= 3.1	0.02	#				
					(5m)		First 10<br>Last 10	2.65<br>1.06	0.36<br>0.18									
					(1f)		First 10<br>Last 10	1.54<br>1.11										
	**D**	Marble interacti on	Glut		6	first 10 interactions - z-score peak	Baseline<br>Pre-event<br>Event	-0.14<br>1.51<br>1.43	0.28<br>0.31<br>0.28	RM 1-way ANOVA	time period	F(2,5)=12.0	0.01	*	Tukey	baseline vs. pre-event<br>baseline vs. event<br>pre-event vs. event	0.04<br>0.03<br>0.8	*<br>*<br>ns
					(4m)		Baseline<br>Pre-event<br>Event	-0.21<br>1.91<br>1.84	0.3<br>0.27<br>0.16									
					(2f)		Baseline<br>Pre-event<br>Event	-0.01<br>0.7<br>0.61	0.77<br>0.02<br>0.12									
					6	last 10 interactions - z-score peak	Baseline<br>Pre-event<br>Event	-0.62<br>0.71<br>0.39	0.07<br>0.26<br>0.42	RM 1-way ANOVA	time period	F(2,5)= 7.5	0.02	*	Tukey	baseline vs. pre-event<br>baseline vs. event<br>pre-event vs. event	0.01<br>0.2<br>0.5	*<br>ns<br>ns
					(4m)		Baseline<br>Pre-event<br>Event	-0.70<br>1.0<br>1.04	0.07<br>0.09<br>0.12									
					(2f)		Baseline<br>Pre-event<br>Event	-0.46<br>0.14<br>-0.93	0.06<br>0.68<br>0.09									
					6	z-score peak	First 10<br>Last 10	1.43<br>0.39	0.28<br>0.42	two-tailed paired *t-test*		t(5)=5.0	0.004	##				
					(4m)		First 10<br>Last 10	1.84<br>1.04	0.16<br>0.12									
					(2f)		First 10<br>Last 10	0.61<br>-0.93	0.12<br>0.09									
	**E**	Milk licking	GABA		5	20+s ILI – z-score peak	Baseline<br>Pre-event<br>Event	0.15<br>2.05<br>2.91	0.29<br>0.28<br>0.39	RM 1-way ANOVA	time period	F(2,4)=19.0	0.0009	***	Tukey	baseline vs. pre-event<br>baseline vs. event<br>pre-event vs. event	0.03<br>0.008<br>0.3	*<br>**<br>ns

					(3m)		Baseline<br>Pre-event<br>Event	0.21<br>1.96<br>3.45	0.41<br>0.47<br>0.35									
					(2f)		Baseline<br>Pre-event<br>Event	0.06<br>2.19<br>2.09	0.57<br>0.28<br>0.04									
					5	z-score peak	1-2s ILI<br>2-5s ILI<br>5-10s ILI<br>10-20s ILI<br>20+s ILI	0.47<br>0.94<br>1.88<br>2.68<br>2.91	0.06<br>0.09<br>0.31<br>0.28<br>0.39	RM 1-way ANOVA	ILI	F(4,4)= 16.33	0.005	**				
					(3m)		1-2s ILI<br>2-5s ILI<br>5-10s ILI<br>10-20s ILI<br>20+s ILI	0.43<br>0.89<br>1.49<br>2.3<br>3.45	0.1<br>0.11<br>0.31<br>0.22<br>0.35									
					(2f)		1-2s ILI<br>2-5s ILI<br>5-10s ILI<br>10-20s ILI<br>20+s ILI	0.55<br>1.02<br>2.47<br>3.24<br>2.09	0.07<br>0.19<br>0.37<br>0.37<br>0.04									
					5	z-score peak	ILI	R^2 = 0.6		linear regression		F(1,23)= 34.8	<0.0001	****				
					Animal 1(m)			R^2 = 0.95		linear regression		F(1,3)= 51.9	0.005	**				
					Animal 2(m)			R^2 = 0.95		linear regression		F(1,3)= 58.5	0.005	**				
					Animal 3(m)			R^2 = 0.98		linear regression		F(1,3)= 174.8	0.001	**				
					Animal 4(f)			R^2 = 0.3		linear regression		F(1,3)= 1.2	0.4	ns				
					Animal 5(f)			R^2 = 0.24		linear regression		F(1,3)= 0.96	0.4	ns				
	**F**	Milk licking	Glut		4	20+s ILI - z-score peak	Baseline<br>Pre-event<br>Event	0.39<br>2.41<br>3.32	0.37<br>0.25<br>0.57	RM 1-way ANOVA	time period	F(2,3)=20.5	0.01	*	Tukey	baseline vs. pre-event<br>baseline vs. event<br>pre-event vs. event	0.01<br>0.01<br>0.4	*<br>*<br>ns
					(2m)		Baseline<br>Pre-event<br>Event	0.8<br>2.4<br>4.3	0.01<br>0.42<br>0.04									
					(2f)		Baseline<br>Pre-event<br>Event	-0.02<br>2.42<br>2.34	0.7<br>0.43<br>0.09									
					4	z-score peak	1-2s ILI<br>2-5s ILI<br>5-10s ILI<br>10-20s ILI<br>20+s ILI	0.44<br>1.3<br>1.48<br>2.58<br>3.32	0.31<br>0.07<br>0.5<br>0.46<br>0.57	RM 1-way ANOVA	ILI	F(4,3)=6.4	0.04	*				
					(2m)		1-2s ILI<br>2-5s ILI<br>5-10s ILI<br>10-20s ILI<br>20+s ILI	0.06<br>1.24<br>0.92<br>2.81<br>4.3	0.53<br>0.07<br>0.34<br>0.46<br>0.04									
					(2f)		1-2s ILI<br>2-5s ILI<br>5-10s ILI<br>10-20s ILI<br>20+s ILI	0.82<br>1.36<br>2.04<br>2.34<br>2.34	0.11<br>0.12<br>0.86<br>0.99<br>0.09									

					4	z-score peak	ILI	R^2 = 0.59		linear regression		F(1,18)= 26.01	<0.0001	****				
					Animal 1(m)			R^2 = 0.97		linear regression		F(1,3)= 108.5	0.002	**				
					Animal 2(m)			R^2 = 0.84		linear regression		F(1,3)= 16	0.03	*				
					Animal 3(f)			R^2 = 0.24		linear regression		F(1,3)= 0.93	0.4	ns				
					Animal 4(f)			R^2 = 0.8		linear regression		F(1,3)= 12.1	0.04	*				
**2**	**A**	Chocolate vs. quinine pellet interaction	GABA		6	Chocolate pellet – z-score peak	Baseline<br>Pre-event<br>Event	0.01<br>1.84<br>2.22	0.38<br>0.31<br>0.31	RM 1-way ANOVA	time period	F(2,5)=13.42	0.01	*	Tukey	baseline vs. pre-event<br>baseline vs. event<br>pre-event vs. event	0.049<br>0.02<br>0.0007	*<br>*<br>***
					(3m)		Baseline<br>Pre-event<br>Event	-0.33<br>1.66<br>2.1	0.39<br>0.55<br>0.59									
					(3f)		Baseline<br>Pre-event<br>Event	0.35<br>2.02<br>2.34	0.67<br>0.36<br>0.32									
					6	Quinine pellet – z-score peak	Baseline<br>Pre-event<br>Event	-0.23<br>0.38<br>1.31	0.32<br>0.49<br>0.14	RM 1-way ANOVA	time period	F(2,5)=4.43	0.06	ns				
					(3m)		Baseline<br>Pre-event<br>Event	-0.26<br>0.24<br>1.23	0.48<br>0.78<br>0.19									
					(3f)		Baseline<br>Pre-event<br>Event	-0.2<br>0.53<br>1.39	0.55<br>0.77<br>0.23									
					6	z-score peak	chocolate<br>qunine	2.22<br>1.31	0.31<br>0.14	two-tailed paired *t-test*		t(5)= 4.55	0.006	##				
					(3m)		chocolate<br>qunine	2.1<br>1.23	0.59<br>0.19									
					(3f)		chocolate<br>qunine	2.34<br>1.39	0.32<br>0.23									
	**B**	Chocolate vs. quinine pellet interaction	Glut		6	Chocolate pellet – z-score peak	Baseline<br>Pre-event<br>Event	-0.20<br>2.21<br>2.29	0.26<br>0.54<br>0.55	RM 1-way ANOVA	time period	F(2,5)=10.94	0.02	*	Tukey	baseline vs. pre-event<br>baseline vs. event<br>pre-event vs. event	0.048<br>0.046<br>0.2	*<br>*<br>ns
					(3m)		Baseline<br>Pre-event<br>Event	-0.65<br>2.92<br>2.98	0.18<br>0.92<br>0.99									
					(3f)		Baseline<br>Pre-event<br>Event	0.25<br>1.51<br>1.59	0.32<br>0.31<br>0.29									
					6	Quinine pellet – z-score peak	Baseline<br>Pre-event<br>Event	-0.63<br>1.78<br>1.73	0.20<br>0.35<br>0.47	RM 1-way ANOVA	time period	F(2,5) = 20.77	0.005	**	Tukey	baseline vs. pre-event<br>baseline vs. event<br>pre-event vs. event	0.008<br>0.02<br>0.9	**<br>*<br>ns
					(3m)		Baseline<br>Pre-event<br>Event	-0.76<br>2.46<br>2.62	0.04<br>0.39<br>0.56									

					(3f)		Baseline<br>Pre-event<br>Event	-0.49<br>1.11<br>0.84	0.43<br>0.13<br>0.04									
					6	z-score peak	chocolate<br>qunine	2.29<br>1.73	0.55<br>0.47	two-tailed paired *t-test*		t(5) = 0.88	0.4	ns				
					(3m)		chocolate<br>qunine	2.98<br>2.62	0.99<br>0.56									
					(3f)		chocolate<br>qunine	1.59<br>0.84	0.29<br>0.04									
	**C**	Conditioned shock	GABA		5	z-score peak	cue - conditioning<br>cue - extinction<br>shock - conditioning<br>shock - extinction	3.12<br>4.36<br>12.36<br>1.76	0.68<br>0.65<br>2.57<br>0.73	RM 2-way ANOVA	stimulus<br>session<br>interaction	F (1, 4) = 4.501<br>F (1, 4) = 12.23<br>F (1, 4) = 23.95	0.1<br>0.03<br>0.008	ns<br>*<br>**	Sidak	conditioning vs. extinction - cue<br>conditioning vs. extinction - shock	0.8<br>0.007	ns<br>&&
					(5m)		cue - conditioning<br>cue - extinction<br>shock - conditioning<br>shock - extinction	3.12<br>4.36<br>12.36<br>1.76	0.68<br>0.65<br>2.57<br>0.73									
					(0f)		cue - conditioning<br>cue - extinction<br>shock - conditioning<br>shock - extinction											
	**D**	Conditioned shock	Glut		4	z-score peak	cue - conditioning<br>cue - extinction<br>shock - conditioning<br>shock - extinction	0.09<br>-0.51<br>3.32<br>-0.43	0.47<br>0.41<br>0.65<br>0.33	RM 2-way ANOVA	stimulus<br>session<br>interaction	F (1, 3) = 6.54<br>F (1, 3) = 68.18<br>F (1, 3) = 13.25	0.08<br>0.004<br>0.04	ns<br>**<br>*	Sidak	conditioning vs. extinction - cue<br>conditioning vs. extinction - shock	0.6<br>0.02	ns<br>&
					(4m)		cue - conditioning<br>cue - extinction<br>shock - conditioning<br>shock - extinction	0.09<br>-0.51<br>3.32<br>-0.43	0.47<br>0.41<br>0.65<br>0.33									
					(0f)		cue - conditioning<br>cue - extinction<br>shock - conditioning<br>shock - extinction											
**3**	**B**	RTPP	GABA	DA	5	Time in paired side	pre-test<br>test 1<br>test 2<br>test 3<br>test 4<br>test 5<br>test 6 (test day)	48.21<br>90.22<br>88.43<br>13.69<br>10.65<br>83.28<br>22.55	4.09<br>4.76<br>3.91<br>1.86<br>2.77<br>0.79<br>5.73	RM 2-way ANOVA	group<br>days<br>interaction	F (2, 15) = 0.3<br>F (6, 90) = 389.4<br>F (12, 90) = 2.7	0.7<br><0.0001<br>0.004	ns<br>****<br>**	Sidak	pre-test vs. test 1<br>pre-test vs. test 2<br>pre-test vs. test 3<br>pre-test vs. test 4<br>pre-test vs. test 5<br>pre-test vs. test 6	<0.0001<br><0.0001<br><0.0001<br><0.0001<br><0.0001<br><0.0001	&&&&<br>&&&&<br>&&&&<br>&&&&<br>&&&&<br>&&&&
					(2m)			41.75<br>83.21<br>83.15<br>15.75	7.97<br>11.16<br>6.80<br>1.69									

								13.36<br>82.25<br>28.81	5.32<br>0.56<br>16.09									
					(3f)			52.51<br>94.90<br>91.95<br>12.32<br>8.85<br>83.96<br>18.38	3.37<br>2.58<br>4.47<br>2.88<br>3.49<br>1.18<br>1.12									
			GABA	Glut	8		pre-test<br>test 1<br>test 2<br>test 3<br>test 4<br>test 5<br>test 6	50.61<br>93.03<br>94.11<br>8.10<br>6.93<br>90.54<br>9.10	3.51<br>1.68<br>1.28<br>1.85<br>1.51<br>1.78<br>1.76							pre-test vs. test 1<br>pre-test vs. test 2<br>pre-test vs. test 3<br>pre-test vs. test 4<br>pre-test vs. test 5<br>pre-test vs. test 6	<0.0001<br><0.0001<br><0.0001<br><0.0001<br><0.0001<br><0.0001	&&&&<br>&&&&<br>&&&&<br>&&&&<br>&&&&<br>&&&&
					(3m)			56.53<br>95.03<br>95.73<br>5.93<br>5.13<br>90.43<br>8.43	3.22<br>1.82<br>2.78<br>0.60<br>0.37<br>3.04<br>3.13									
					(5f)			47.06<br>91.82<br>93.14<br>9.40<br>8.00<br>90.60<br>9.50	4.79<br>2.43<br>1.29<br>2.89<br>2.36<br>2.46<br>2.38									
			GABA	GABA	5		pre-test<br>test 1<br>test 2<br>test 3<br>test 4<br>test 5<br>test 6	48.80<br>85.60<br>85.80<br>14.00<br>14.20<br>76.60<br>20.00	7.19<br>4.26<br>4.65<br>4.95<br>4.84<br>2.94<br>3.75							pre-test vs. test 1<br>pre-test vs. test 2<br>pre-test vs. test 3<br>pre-test vs. test 4<br>pre-test vs. test 5<br>pre-test vs. test 6	<0.0001<br><0.0001<br><0.0001<br><0.0001<br><0.0001<br><0.0001	&&&&<br>&&&&<br>&&&&<br>&&&&<br>&&&&<br>&&&&
					(2m)			50.50<br>95.00<br>97.00<br>2.00<br>3.00<br>80.50<br>12.50	22.50<br>2.00<br>1.00<br>2.00<br>3.00<br>0.50<br>4.50									
					(3f)			47.67<br>79.33<br>78.33<br>22.00<br>21.67<br>74.00<br>25.00	1.33<br>3.18<br>1.45<br>0.58<br>2.33<br>4.51<br>3.00									
	**D**	Passive<br>stim<br>5s	GABA	DA	5	z-score<br>peak	pre-<br>early-<br>late-<br>stimulation	-0.11<br>18.13<br>17.48	0.32<br>2.69<br>2.23	RM 1-<br>way<br>ANOVA	time<br>period	F(2,4)=4<br>1.37	0.001	**	Tukey	pre vs. early<br>pre vs. late<br>early vs. late	0.007<br>0.004<br>0.9	**<br>**<br>ns
					(2m)			0.17<br>14.29<br>13.25	0.65<br>5.82<br>3.74									
					(3f)			-0.30<br>20.69<br>20.31	0.39<br>2.14<br>1.40									
	**E**	Passive<br>stim<br>5s	GABA	Glut	8	z-score<br>peak	pre-<br>early-<br>late-<br>stimulation	0.48<br>7.87<br>6.84	0.16<br>1.01<br>1.37	RM 1-<br>way<br>ANOVA	time<br>period	F(2,7)=2<br>7.81	0.0001	***	Tukey	pre vs. early<br>pre vs. late<br>early vs. late	0.0003<br>0.005<br>0.5	***<br>**<br>ns

					(3m)			0.81<br>10.44<br>8.51	0.11<br>0.21<br>1.42									
					(5f)			0.29<br>6.34<br>5.84	0.21<br>1.11<br>2.00									
	**F**	Passive<br>stim<br>5s	GABA	GABA	5	z-score<br>peak	pre-<br>early-<br>late-<br>stimulation	0.18<br>3.17<br>-2.82	0.37<br>1.76<br>0.50	RM 1-<br>way<br>ANOVA	time<br>period	F(2,4)=9.<br>37	0.04	*	Tukey	pre vs. early<br>pre vs. late<br>early vs. late	0.3<br>0.0001<br>0.05	ns<br>***<br>ns
					(2m)			0.83<br>5.72<br>-2.11	0.07<br>3.29<br>0.18									
					(3f)			-0.25<br>1.48<br>-3.29	0.48<br>1.78<br>0.74									
	**H**	RTPP<br>2s off /<br>5s on	GABA	DA	5	z-score<br>peak	pre-<br>early-<br>late-<br>stimulation	-0.61<br>7.93<br>3.18	0.25<br>1.23<br>0.34	RM 1-<br>way<br>ANOVA	time<br>period	F(2,4)=3<br>1.45	0.003	**	Tukey	pre vs. early<br>pre vs. late<br>early vs. late	0.007<br>0.001<br>0.04	**<br>**<br>*
					(2m)			-0.21<br>8.82<br>3.51	0.56<br>3.49<br>0.14									
					(3f)			-0.88<br>7.33<br>2.96	0.09<br>0.70<br>0.57									
	**I**	RTPP<br>2s off /<br>5s on	GABA	Glut	8	z-score<br>peak	pre-<br>early-<br>late-<br>stimulation	0.31<br>3.67<br>0.36	0.28<br>0.55<br>0.73	RM 1-<br>way<br>ANOVA	time<br>period	F(2,7)=1<br>7.37	0.0003	***	Tukey	pre vs. early<br>pre vs. late<br>early vs. late	0.001<br>1.0<br>0.002	**<br>ns<br>**
					(3m)			0.70<br>4.41<br>0.80	0.61<br>1.29<br>1.81									
					(5f)			0.08<br>3.22<br>0.09	0.27<br>0.48<br>0.68									
	**J**	RTPP<br>2s off /<br>5s on	GABA	GABA	5	z-score<br>peak	pre-<br>early-<br>late-<br>stimulation	0.51<br>-1.31<br>-2.04	0.08<br>0.25<br>0.26	RM 1-<br>way<br>ANOVA	Passive<br>stim 1s	F(2,4)=6<br>4.96	< 0.0001	****	Tukey	pre vs. early<br>pre vs. late<br>early vs. late	0.003<br>0.001<br>0.04	**<br>**<br>*
					(2m)			0.63<br>-1.15<br>-2.28	0.01<br>0.34<br>0.11									
					(3f)			0.43<br>-1.41<br>-1.87	0.11<br>0.39<br>0.44									
	**K**	Passive<br>stim 5s<br>vs.<br>RTPP<br>2s off /<br>5s on	GABA	DA	5	z-score<br>peak	passive - early<br>RTPP - early<br>passive - late<br>RTPP - late	18.13<br>7.93<br>17.48<br>3.18	2.68<br>1.23<br>2.23<br>0.34	RM 2-<br>way<br>ANOVA	time<br>test type<br>interaction	F (1, 4) =<br>8.37<br>F (1, 4) =<br>30.51<br>F (1, 4) =<br>6.17	0.04<br>0.005<br>0.07	*<br>**<br>ns	Sidak	passive vs. RTPP - early<br>passive vs. RTPP - late	0.002<br>0.0005	&&<br>&&&
					(2m)			14.29<br>8.82<br>13.25<br>3.51	2.69<br>3.49<br>3.74<br>0.14									
					(3f)			20.69<br>7.33<br>20.31<br>2.96	2.14<br>0.70<br>1.40<br>0.57									
	**L**	Passive<br>stim 5s<br>vs.<br>RTPP	GABA	Glut	8	z-score<br>peak	passive - early<br>RTPP - early<br>passive - late<br>RTPP - late	7.87<br>3.67<br>6.84<br>0.36	1.01<br>0.55<br>1.37<br>0.73	RM 2-<br>way<br>ANOVA	time<br>test type<br>interaction	F (1, 7) =<br>12.59<br>F (1, 7) =<br>38.28	0.009<br>0.0005<br>0.04	**<br>***<br>*	Sidak	passive vs. RTPP - early<br>passive vs. RTPP - late	0.0006<br>< 0.0001	&&&<br>&&&&

		2s off /<br>5s on										F (1, 7) =<br>6.46						
					(3m)			10.44<br>4.41<br>8.51<br>0.80	0.21<br>1.29<br>1.42<br>1.81									
					(5f)			6.34<br>3.22<br>5.84<br>0.09	1.11<br>0.48<br>2.00<br>0.68									
	**M**	Passive<br>stim 5s<br>vs.<br>RTPP<br>2s off /<br>5s on	GABA	GABA	5	z-score<br>peak	passive - early<br>RTPP - early<br>passive - late<br>RTPP - late	3.17<br>-1.31<br>-2.82<br>-2.04	1.76<br>0.25<br>0.50<br>0.26	RM 2-<br>way<br>ANOVA	time<br>test type<br>interaction	F (1, 4) =<br>13.71<br>F (1, 4) =<br>4.77<br>F (1, 4) =<br>10.16	0.02<br>0.09<br>0.03	*<br>ns<br>*	Sidak	passive vs. RTPP - early<br>passive vs. RTPP - late	0.04<br>0.8	&<br>ns
					(2m)			5.72<br>-1.15<br>-2.11<br>-2.28	3.29<br>0.34<br>0.18<br>0.11									
					(3f)			1.48<br>-1.41<br>-3.29<br>-1.87	1.78<br>0.39<br>0.74<br>0.44									
**4**	**B**	Passive<br>stim 1s,<br>ICSS,<br>PPB	GABA	DA	5	Event<br>–<br>z-score<br>peak	Passive stim<br>ICSS<br>PPB	14.62<br>6.08<br>10.06	2.36<br>1.61<br>2.10	RM 1-<br>way<br>ANOVA	assay<br>type	F(2,4)=<br>28.93	0.0009	***	Tukey	passive stim vs. ICSS<br>passive stim vs. PPB<br>ICSS vs. PPB	0.007<br>0.03<br>0.02	**<br>*<br>*
					(2m)			13.86<br>4.90<br>10.12	6.36<br>3.16<br>4.21									
					(3f)			15.13<br>6.87<br>10.01	2.19<br>2.13<br>2.96									
	**C**	Passive<br>stim 1s,<br>ICSS,<br>PPB,<br>Nosepo<br>ke PPB	GABA	DA	5	Pre-event –<br>z-score<br>peak	Passive stim<br>ICSS<br>PPB<br>Nosepoke PPB	-0.09<br>0.22<br>-0.22<br>0.30	0.23<br>0.11<br>0.01<br>0.14	RM 1-<br>way<br>ANOVA	assay<br>type	F(3,4)=<br>2.31	0.2	ns				
					(2m)			0.48<br>0.02<br>-0.25<br>0.09	0.02<br>0.20<br>0.01<br>0.28									
					(3f)			-0.46<br>0.35<br>-0.20<br>0.44	0.05<br>0.10<br>0.01<br>0.12									
	**D**	Passive<br>stim 1s,<br>ICSS,<br>PPB	GABA	Glut	8	Event<br>–<br>z-score<br>peak	Passive stim<br>ICSS<br>PPB	6.17<br>4.14<br>6.46	0.73<br>0.50<br>0.65	RM 1-<br>way<br>ANOVA	assay<br>type	F(2,7)=<br>9.57	0.009	**	Tukey	passive stim vs. ICSS<br>passive stim vs. PPB<br>ICSS vs. PPB	0.07<br>0.8<br>0.003	ns<br>ns<br>**
					(3m)			7.54<br>5.24<br>8.43	0.70<br>0.40<br>0.39									
					(5f)			5.35<br>3.47<br>5.27	0.94<br>0.59<br>0.47									
	**E**	Passive<br>stim 1s,<br>ICSS,<br>PPB,<br>Nosepo<br>ke PPB	GABA	Glut	8	Pre-event –<br>z-score<br>peak	Passive stim<br>ICSS<br>PPB<br>Nosepoke PPB	-0.05<br>0.39<br>-0.03<br>0.75	0.27<br>0.21<br>0.12<br>0.12	RM 1-<br>way<br>ANOVA	assay<br>type	F(3,7)=<br>3.9	0.06	ns				

					(3m)			-0.30<br>0.25<br>-0.05<br>0.62	0.58<br>0.21<br>0.17<br>0.08									
					(5f)			0.10<br>0.47<br>-0.02<br>0.82	0.31<br>0.32<br>0.18<br>0.19									
	**F**	Passive stim 1s, ICSS, PPB	GABA	GABA	5	Event – z-score peak	Passive - max<br>ICSS - max<br>PPB max<br>Passive - min<br>ICSS - min<br>PPB min	3.09<br>2.60<br>2.48<br>-1.27<br>-0.75<br>-0.73	0.86<br>0.48<br>0.73<br>0.32<br>0.29<br>0.30	RM 2-way ANOVA	assay<br>peak type<br>interaction	F(2,8)= 0.004<br>F(1,4)= 101.5<br>F(2,8)= 0.75	1.0<br>0.0005<br>0.5	ns<br>***<br>ns	Sidak	passive vs. ICSS - max<br>passive vs. PPB - max<br>ICSS vs. PPB - max<br>passive vs. ICSS - min<br>passive vs. PPB - min<br>ICSS vs. PPB - min	0.9<br>0.8<br>1.0<br>0.9<br>0.9<br>> 1.0	ns<br>ns<br>ns<br>ns<br>ns<br>ns
					(2m)			4.64<br>3.12<br>1.64<br>-0.82<br>-0.25<br>-0.45	1.64<br>1.17<br>0.36<br>0.19<br>0.15<br>0.37									
					(3f)			2.05<br>2.25<br>3.04<br>-1.56<br>-1.09<br>-0.92	0.47<br>0.40<br>1.16<br>0.46<br>0.37<br>0.46									
	**G**	Passive stim 1s, ICSS, PPB, Nosepoke PPB	GABA	GABA	5	Pre-event – z-score peak	Passive stim<br>ICSS<br>PPB<br>Nosepoke PPB	-0.23<br>1.72<br>-0.21<br>1.54	0.37<br>0.28<br>0.11<br>0.35	RM 1-way ANOVA	assay type	F(3,4)= 16.59	0.002	**	Tukey	passive vs. ICSS<br>passive vs. PPB<br>passive vs. nosepoke PPB<br>ICSS vs. PPB<br>ICSS vs. nosepoke PPB<br>PPB vs. nosepoke PPB	0.03<br>> 1.0<br>0.07<br>0.02<br>0.8<br>0.05	*<br>ns<br>ns<br>*<br>ns<br>*
					(2m)			0.67<br>1.81<br>-0.03<br>1.59	0.19<br>0.83<br>0.22<br>0.87									
					(3f)			-0.83<br>1.66<br>-0.34<br>1.51	0.06<br>0.17<br>0.05<br>0.38									
**5**	**B**	RTPP	Glut	DA	6	Time in paired side	pre-test<br>test 1<br>test 2<br>test 3<br>test 4<br>test 5<br>test 6<br>(test day)	51.88<br>4.22<br>1.59<br>79.74<br>94.30<br>2.60<br>96.41	7.62<br>1.21<br>0.64<br>12.67<br>2.57<br>0.62<br>1.69	RM 2-way ANOVA	group<br>days<br>interaction	F (2, 16) = 1.09<br>F (6, 96) = 265.6<br>F (12, 96) = 0.42	0.4<br>< 0.0001<br>1.0	ns<br>****<br>ns	Sidak	pre-test vs. test 1<br>pre-test vs. test 2<br>pre-test vs. test 3<br>pre-test vs. test 4<br>pre-test vs. test 5<br>pre-test vs. test 6	< 0.0001<br>< 0.0001<br>0.0005<br>< 0.0001<br>< 0.0001<br>< 0.0001	&&&&<br>&&&&<br>&&&<br>&&&&<br>&&&&<br>&&&&
					(3m)			57.68<br>4.38<br>0.50<br>63.45<br>93.03<br>2.28<br>97.87	10.24<br>2.39<br>0.13<br>23.14<br>5.15<br>1.24<br>1.25									
					(3f)			46.07<br>4.05<br>2.68<br>96.03<br>95.56<br>2.92<br>94.95	12.32<br>1.27<br>0.92<br>1.20<br>2.18<br>0.51<br>3.26									
				Glut	7		pre-test<br>test 1	50.93<br>9.55	7.56<br>4.16							pre-test vs. test 1<br>pre-test vs. test 2	< 0.0001<br>< 0.0001	&&&&<br>&&&&

							test 2<br>test 3<br>test 4<br>test 5<br>test 6	3.61<br>94.66<br>95.10<br>6.43<br>96.05	2.00<br>2.17<br>2.27<br>3.41<br>1.98							pre-test vs. test 3<br>pre-test vs. test 4<br>pre-test vs. test 5<br>pre-test vs. test 6	< 0.0001<br>< 0.0001<br>< 0.0001<br>< 0.0001	&&&&<br>&&&&<br>&&&&<br>&&&&
					(3m)			46.51<br>11.97<br>2.75<br>95.05<br>97.70<br>8.94<br>96.96	13.77<br>10.00<br>2.41<br>3.44<br>1.90<br>8.16<br>1.92									
					(4f)			54.24<br>7.74<br>4.26<br>94.38<br>93.16<br>4.56<br>95.37	9.84<br>2.85<br>3.29<br>3.25<br>3.64<br>2.14<br>3.40									
				GABA	6		pre-test<br>test 1<br>test 2<br>test 3<br>test 4<br>test 5<br>test 6	53.33<br>8.17<br>7.83<br>87.17<br>94.83<br>4.17<br>95.17	7.72<br>1.74<br>2.54<br>7.73<br>2.30<br>1.19<br>1.89							pre-test vs. test 1<br>pre-test vs. test 2<br>pre-test vs. test 3<br>pre-test vs. test 4<br>pre-test vs. test 5<br>pre-test vs. test 6	< 0.0001<br>< 0.0001<br>< 0.0001<br>< 0.0001<br>< 0.0001<br>< 0.0001	&&&&<br>&&&&<br>&&&&<br>&&&&<br>&&&&<br>&&&&
					(3m)			49.33<br>7.33<br>8.67<br>81.67<br>97.67<br>3.00<br>97.00	15.84<br>1.20<br>4.37<br>15.90<br>1.45<br>1.00<br>1.53									
					(3f)			57.33<br>9.00<br>7.00<br>92.67<br>92.00<br>5.33<br>93.33	5.61<br>3.61<br>3.51<br>3.93<br>4.04<br>2.19<br>3.48									
	**D**	Passive stim 1s	Glut	DA	6	z-score peak	Pre-stim<br>Stim	-0.38<br>4.25	0.24<br>0.27	two-tailed paired *t-test*		t(5)= 13.75	<0.0001	####				
					(3m)			-0.70<br>4.27	0.09<br>0.42									
					(3f)			-0.07<br>4.23	0.42<br>0.42									
		Passive stim 5s			6		Pre-<br>Early-<br>Late-<br>Stimulation	-0.53<br>2.26<br>1.73	0.07<br>0.19<br>0.30	RM 1-way ANOVA	time period	F(2,5)= 42.7	0.0001	***	Tukey	pre vs. early<br>pre vs. late<br>early vs. late	0.0001<br>0.003<br>0.4	***<br>**<br>ns
					(3m)			-0.62<br>2.41<br>2.24	0.09<br>0.36<br>0.35									
					(3f)			-0.45<br>2.11<br>1.22	0.10<br>0.15<br>0.29									
	**E**	Passive stim 1s	Glut	Glut	7	z-score peak	Pre-stim<br>Stim	-0.29<br>6.67	0.29<br>0.92	two-tailed paired *t-test*		t(6)= 7.8	0.0002	###				
					(3m)			-0.80<br>4.86	0.10<br>0.79									
					(4f)			0.10<br>8.02	0.41<br>1.10									

		Passive<br>stim 5s			7		Pre-<br>Early-<br>Late-<br>Stimulation	0.35<br>5.64<br>5.23	0.28<br>0.56<br>0.48	RM 1-<br>way<br>ANOVA	time<br>period	F(2,6)=<br>50.48	< 0.0001	****	Tukey	pre vs. early<br>pre vs. late<br>early vs. late	0.0006<br>0.0006<br>0.6	***<br>***<br>ns
					(3m)			0.78<br>5.01<br>4.13	0.13<br>0.83<br>0.70									
					(4f)			0.02<br>6.11<br>6.06	0.43<br>0.75<br>0.19									
	**F**	Passive<br>stim 1s	Glut	GABA	6	z-score<br>peak	Pre-stim<br>Stim	-0.37<br>6.88	0.26<br>1.28	two-<br>tailed<br>paired<br>*t-test*		t(5)=<br>5.92	0.002	##				
					(3m)			-0.65<br>5.01	0.07<br>2.10									
					(3f)			-0.10<br>8.75	0.50<br>0.49									
		Passive<br>stim 5s			6		Pre-<br>Early-<br>Late-<br>Stimulation	-0.16<br>7.77<br>10.36	0.32<br>0.70<br>1.37	RM 1-<br>way<br>ANOVA	time<br>period	F(2,5)=<br>73.89	0.0002	***	Tukey	pre vs. early<br>pre vs. late<br>early vs. late	< 0.0001<br>0.0009<br>0.05	****<br>***<br>ns
					(3m)			-0.20<br>7.72<br>11.42	0.47<br>1.41<br>2.55									
					(3f)			-0.12<br>7.82<br>9.29	0.54<br>0.67<br>1.35									
	**G**	RTPP<br>2s off /<br>1s on	Glut	DA	6	z-score<br>peak	Pre-stim<br>Stim	0.38<br>2.52	0.49<br>0.23	two-<br>tailed<br>paired<br>*t-test*		t(5)=<br>3.84	0.01	#				
					(3m)			0.56<br>2.14	0.78<br>0.26									
					(3f)			0.21<br>2.91	0.75<br>0.22									
	**H**	RTPP<br>2s off /<br>1s on	Glut	Glut	7	z-score<br>peak	Pre-stim<br>Stim	0.66<br>5.41	0.57<br>0.81	two-<br>tailed<br>paired<br>*t-test*		t(6)=<br>8.41	0.0002	###				
					(3m)			1.21<br>5.69	1.15<br>1.51									
					(4f)			0.25<br>5.21	0.58<br>1.07									
	**I**	RTPP<br>2s off /<br>1s on	Glut	GABA	6	z-score<br>peak	Pre-stim<br>Stim	0.02<br>4.91	0.40<br>1.23	two-<br>tailed<br>paired<br>*t-test*		t(5)=<br>4.652	0.006	##				
					(3m)			-0.02<br>3.59	0.46<br>1.94									
					(3f)		[[[p[	0.07<br>6.23	0.76<br>1.42									
**6**	**A**<br>**1**	ICSS<br>vs.<br>PPB	GABA	DLight<br>-<br>NAc<br>dorsal<br>msh	6	z-score<br>peak	ICSS<br>PPB	3.85<br>5.98	0.53<br>0.71	two-<br>tailed<br>paired<br>*t-test*		t(5)=<br>5.23	0.003	##				
					(3m)			4.16<br>6.25	0.87<br>0.85									
					(3f)			3.53<br>5.71	0.75<br>1.30									
	**A**<br>**2**	RTPP<br>2s off/<br>5s on	GABA	DLight<br>-	6	z-score<br>peak	RTPP - early<br>Passive - early<br>RTPP - late	3.47<br>6.23<br>1.05	0.60<br>0.74<br>0.20	RM 2-<br>way<br>ANOVA	time<br>assay<br>interaction	F (1, 5) =<br>5.68	0.06<br>0.003<br>0.002	ns<br>**<br>**	Sidak	RTPP vs. passive - early<br>RTPP vs. passive - late	0.0004<br>< 0.0001	&&&<br>&&&&

		vs.<br>Passive<br>stim 5s		NAc<br>dorsal<br>msh			Passive - late	6.07	0.81			F (1, 5) =<br>30.99<br>F (1, 5) =<br>31.74						
					(3m)			4.19<br>6.58<br>1.17<br>5.48	1.09<br>1.09<br>0.39<br>0.12									
					(3f)			2.76<br>5.89<br>0.92<br>6.66	0.37<br>1.19<br>0.18<br>1.72									
	**B<br>1**	ICSS<br>vs.<br>PPB	GABA	DLight<br>-<br>NAc<br>ventra<br>l msh	5	z-score<br>peak	ICSS<br>PPB	5.65<br>10.35	0.70<br>1.70	two-<br>tailed<br>paired<br>*t-test*		t(4)=<br>3.92	0.02	#				
					(2m)			5.51<br>11.54	1.09<br>4.16									
					(3f)			5.74<br>9.55	1.11<br>1.76									
	**B<br>2**	RTPP<br>2s off/<br>5s on<br>vs.<br>Passive<br>stim 5s	GABA	DLight<br>-<br>NAc<br>ventra<br>l msh	5	z-score<br>peak	RTPP - early<br>Passive - early<br>RTPP - late<br>Passive - late	5.12<br>9.11<br>1.44<br>8.19	0.60<br>1.41<br>0.17<br>1.88	RM 2-<br>way<br>ANOVA	time<br>assay<br>interaction	F (1, 4) =<br>9.13<br>F (1, 5) =<br>10.42<br>F (1, 5) =<br>31.6	0.04<br>0.03<br>0.005	*<br>*<br>**	Sidak	RTPP vs. passive - early<br>RTPP vs. passive - late	0.0007<br>< 0.0001	&&&<br>&&&&
					(2m)			5.39<br>9.63<br>1.23<br>7.42	0.22<br>4.01<br>0.30<br>5.23									
					(3f)			4.94<br>8.76<br>1.57<br>8.70	1.07<br>1.05<br>0.21<br>1.54									
	**C<br>1**	Passive<br>stim 1s	Glut	DLight<br>-<br>NAc<br>dorsal<br>msh	5	z-score<br>peak	Pre-stim<br>Stim	-0.17<br>-1.77	0.26<br>0.35	one-<br>tailed<br>paired<br>*t-test*		t(4)=<br>2.71	0.03	#				
					(4m)			-0.02<br>-1.98	0.28<br>0.35									
					(1f)			-0.73<br>-0.93										
		RTPP<br>2s off /<br>1s on	Glut	DLight<br>-<br>NAc<br>dorsal<br>msh	5	z-score<br>peak	Pre-stim<br>Stim	0.61<br>-0.91	0.32<br>0.40	one-<br>tailed<br>paired<br>*t-test*		t(4)=<br>2.13	0.049	#				
					(4m)			0.55<br>-0.90	0.41<br>0.51									
					(1f)			0.83<br>-0.96										
	**C<br>2**	Passive<br>stim 5s	Glut	DLight<br>-<br>NAc<br>dorsal<br>msh	5	z-score<br>peak	Pre-<br>Early-<br>Late-<br>Stimulation	-0.14<br>-1.83<br>-2.12	0.28<br>0.31<br>0.48	RM 1-<br>way<br>ANOVA	time<br>period	F(4,8)=1<br>2.62	0.02	*	Holm-<br>Sidak	pre vs. early<br>pre vs. late<br>early vs. late	0.046<br>0.049<br>0.2	*<br>*<br>ns
					(4m)			-0.29<br>-1.85<br>-2.01	0.30<br>0.40<br>0.60									
					(1f)			0.47<br>-1.75<br>-2.54										

	**D<br>1**	Passive<br>stim 1s	Glut	DLight<br>-<br>NAc<br>ventra<br>l msh	4	z-score<br>peak	Pre-stim<br>Stim	0.34<br>1.81	1.81<br>0.45	one-<br>tailed<br>paired<br>*t-test*		t(3)=<br>2.69	0.04	#				
					(2m)			0.75<br>2.22	0.03<br>0.93									
					(2f)			-0.07<br>1.40	0.75<br>0.19									
		RTPP<br>2s off /<br>1s on	Glut	DLight<br>-<br>NAc<br>ventra<br>l msh	4	z-score<br>peak	Pre-stim<br>Stim	0.33<br>2.06	0.33<br>0.27	one-<br>tailed<br>paired<br>*t-test*		t(3)=<br>3.33	0.02	#				
					(2m)			-0.05<br>2.49	0.56<br>0.06									
					(2f)			0.72<br>1.62	0.24<br>0.21									
	**D<br>2**	Passive<br>stim 5s	Glut	DLight<br>-<br>NAc<br>ventra<br>l msh	4	z-score<br>peak	Pre-<br>Early-<br>Late-<br>Stimulation	-0.28<br>1.63<br>2.51	0.38<br>0.41<br>0.31	RM 1-<br>way<br>ANOVA	time<br>period	F(2,3)=<br>20.13	0.002	**	Holm-<br>Sidak	pre vs. early<br>pre vs. late<br>early vs. late	0.01<br>0.002<br>0.1	*<br>**<br>ns
					(2m)			0.17<br>2.18<br>2.81	0.64<br>0.53<br>0.62									
					(2f)			-0.73<br>1.07<br>2.21	0.22<br>0.31<br>0.11									
