# Supplementary material for: Ventral pallidum GABA and glutamate neurons drive approach and avoidance through distinct modulation of VTA cell types
Source: Nat Commun. 2024 May 18;15:4233. doi: 10.1038/s41467-024-48340-y (PMC11102457; doi:10.1038/s41467-024-48340-y)
Supplement: Supplementary file 3 — Reporting Summary [file 41467_2024_48340_MOESM3_ESM.pdf]

nature portfolio

Corresponding author(s): Thomas S. Hnasko

Last updated by author(s): 03/08/2024

Reporting Summary

Nature Portfolio wishes to improve the reproducibility of the work that we publish. This form provides structure for consistency and transparency in reporting. For further information on Nature Portfolio policies, see our Editorial Policies and the Editorial Policy Checklist.

Statistics

For all statistical analyses, confirm that the following items are present in the figure legend, table legend, main text, or Methods section.

n/a	Confirmed	
☐	☒	The exact sample size (*n*) for each experimental group/condition, given as a discrete number and unit of measurement
☐	☒	A statement on whether measurements were taken from distinct samples or whether the same sample was measured repeatedly
☐	☒	The statistical test(s) used AND whether they are one- or two-sided<br>*Only common tests should be described solely by name; describe more complex techniques in the Methods section.*
☐	☒	A description of all covariates tested
☒	☐	A description of any assumptions or corrections, such as tests of normality and adjustment for multiple comparisons
☐	☒	A full description of the statistical parameters including central tendency (e.g. means) or other basic estimates (e.g. regression coefficient) AND variation (e.g. standard deviation) or associated estimates of uncertainty (e.g. confidence intervals)
☐	☒	For null hypothesis testing, the test statistic (e.g. *F*, *t*, *r*) with confidence intervals, effect sizes, degrees of freedom and *P* value noted<br>*Give P values as exact values whenever suitable.*
☒	☐	For Bayesian analysis, information on the choice of priors and Markov chain Monte Carlo settings
☒	☐	For hierarchical and complex designs, identification of the appropriate level for tests and full reporting of outcomes
☐	☒	Estimates of effect sizes (e.g. Cohen's *d*, Pearson's *r*), indicating how they were calculated

Our web collection on statistics for biologists contains articles on many of the points above.

Software and code

Policy information about availability of computer code

Data collection	MED-PC software v.IV (Med Associates) and Any-Maze software v.6 (Stoelting) for behavioral data. Synapse software v.98 and RZ5P processor (Tucker-Davis Technologies) for fiber photometry data. Zen software v.3.5 blue edition, and Zeiss AxioObserver Z1 widefield epifluorescent microscope for histology data. Clampex v.10.6 software (Molecular Devices) for electrophysiological data.
Data analysis	GraphPad Prism v.6 and v.9.2 for statistical analysis. Clampfit v.10.4.2 (Molecular Devices) for electrophysiological data. Mouse Brain Atlas, Paxinos & Franklin, second edition, 2001 version, and Adobe Illustrator v.28.3 for histology maps. MATLAB v.2020a for FP data analysis, with following packages: eeglab v.14.1.1b, raacampbell/shadedErrorBar v.1.62.0.0, chronux v.2.12. Custom-written MATLAB basic codes for FP analysis are available on github at https://github.com/lauren-faget/VPtoVTA_FP.

For manuscripts utilizing custom algorithms or software that are central to the research but not yet described in published literature, software must be made available to editors and reviewers. We strongly encourage code deposition in a community repository (e.g. GitHub). See the Nature Portfolio guidelines for submitting code & software for further information.

Data

Policy information about availability of data

All manuscripts must include a data availability statement. This statement should provide the following information, where applicable:

- Accession codes, unique identifiers, or web links for publicly available datasets
- A description of any restrictions on data availability
- For clinical datasets or third party data, please ensure that the statement adheres to our policy

Source data files are provided with this paper.

Research involving human participants, their data, or biological material

Policy information about studies with human participants or human data. See also policy information about sex, gender (identity/presentation), and sexual orientation and race, ethnicity and racism.

Reporting on sex and gender	n/a
Reporting on race, ethnicity, or other socially relevant groupings	n/a
Population characteristics	n/a
Recruitment	n/a
Ethics oversight	n/a

Note that full information on the approval of the study protocol must also be provided in the manuscript.

Field-specific reporting

Please select the one below that is the best fit for your research. If you are not sure, read the appropriate sections before making your selection.

☒ Life sciences ☐ Behavioural & social sciences ☐ Ecological, evolutionary & environmental sciences

For a reference copy of the document with all sections, see nature.com/documents/nr-reporting-summary-flat.pdf

Life sciences study design

All studies must disclose on these points even when the disclosure is negative.

Sample size	Power analysis were performed a priori or posthoc
Data exclusions	Mice were excluded when misplacement of one of the optic fibers (on top of NAc, VP, or VTA) was detected (number of animals excluded: 6 VGAT-Cre mice, 9 VGLUT2-Cre mice, 3 VGLUT2-Cre; VGAT-FlpO mice).
Replication	When feasible, experiments were validated using multiple approaches. For example, VTA DA neuron activity measurements were performed using calcium and DA sensors. VP cell body GCaMP recordings were performed in at least 2 cohorts of subjects for internal replication. All attempts at replication were successful.
Randomization	Designation of the paired side in the CPP and RTPP assays was pseudo-randomized to obtain ~ 50% preference for each side during pre-test session. No randomization was necessary in the strawberry milk licking, pellet consumption, conditioned footshock, ICSS and passive stimulation assays.
Blinding	Electrophysiological recordings were performed by investigators blind to genotype. Histology was performed blind to photometric or behavioral outcome. Blinding was not relevant for behavioral and fiber photometry assays as only one cohort (identical treatment and genotype) was tested at a time.

Reporting for specific materials, systems and methods

We require information from authors about some types of materials, experimental systems and methods used in many studies. Here, indicate whether each material, system or method listed is relevant to your study. If you are not sure if a list item applies to your research, read the appropriate section before selecting a response.

Materials & experimental systems

n/a	Involved in the study
☐	☒ Antibodies
☒	☐ Eukaryotic cell lines
☒	☐ Palaeontology and archaeology
☐	☒ Animals and other organisms
☒	☐ Clinical data
☒	☐ Dual use research of concern
☒	☐ Plants

Methods

n/a	Involved in the study
☒	☐ ChIP-seq
☒	☐ Flow cytometry
☒	☐ MRI-based neuroimaging

Antibodies

Antibodies used

Primary antibodies:
Rabbit anti-GFP (1:2000; Molecular Probes Cat# A-11122, RRID:AB_221569)
Chicken anti-GFP (1:2000; Thermo Fisher Scientific Cat# A10262, RRID:AB_2534023)
Rabbit anti-TH (1:2000; Millipore Cat# AB152, RRID:AB_390204)
Sheep anti-TH (1:2000; Pel-Freez Biologicals Cat# P60101-0, RRID:AB_461070)
Rat anti-substance P (1:400; Millipore Cat# MAB356, RRID:AB_94639)
Rabbit anti-DsRed (1:2000; Takara Bio Cat# 632496, RRID:AB_10013483)
Secondary antibodies:
All from Jackson ImmunoResearch Labs at 1:400 dilution
Donkey anti-rabbit alexa488 (711-545-152, RRID:AB_2313584)
Donkey anti-chicken alexa488 (703-546-155, RRID:AB_2313584)
Donkey anti-rabbit alexa594 (711-585-152, RRID:AB_2340621)
Donkey anti-rabbit alexa647 (711-605-152, RRID:AB_2492288)
Donkey anti-sheep alexa647 (713-605-147, RRID:AB_2340751)
Donkey anti-rat alexa647 (712-605-153, RRID:AB_2340694)

Validation

All antibodies used are listed on 'The Antibody Registry' website, and RRIDs are provided. Also, macro level images are showing expected patterns, for example GFP and DsRed only in virally-infected areas, TH only in VTA and SNc areas, Substance P in VP area.

Rabbit anti-GFP has been validated by manufacturer using ICC and IF. Relevant citations can be found on manufacturer's website: https://www.thermofisher.com/antibody/product/GFP-Antibody-Polyclonal/A-11122
Chicken anti-GFP has been validated by manufacturer using ICC and IF. Relevant citations can be found on manufacturer's website: https://www.thermofisher.com/antibody/product/GFP-Antibody-Polyclonal/A10262
Rabbit anti-TH has been validated by manufacturer using ELISA, IF, IHC, IP and WB. Relevant citations can be found on manufacturer's website: https://www.emdmillipore.com/US/en/product/Anti-Tyrosine-Hydroxylase-Antibody,MM_NF-AB152?ReferrerURL=https%3A%2F%2Fwww.google.com%2F
Sheep anti-TH has been validated by manufacturer using IHC and WB. Relevant citations can be found on manufacturer's website: https://www.pel-freez.com/p60101-150-ab-sheep-tyrosine-hydroxylase-p60101-150
Rat anti-substance P has been validated by manufacturer using IHC. Relevant citations can be found on manufacturer's website: https://www.emdmillipore.com/US/en/product/Anti-Substance-P-Antibody-pain-clone-NC1,MM_NF-MAB356#documentation
Rabbit anti-DsRed has been validated by manufacturer using WB. Relevant citations can be found on the RRID portal: https://scicrunch.org/resolver/AB_10013483/mentions?q=&i=rrid:ab_10013483-64385

Animals and other research organisms

Policy information about studies involving animals; ARRIVE guidelines recommended for reporting animal research, and Sex and Gender in Research

Laboratory animals

VGLUT2-IRES-Cre, VGAT-IRES-Cre, DAT-IRES-Cre, and VGAT-2A-FlpO-D knock-in mice were obtained from The Jackson Laboratory: Slc17a6tm2(cre)Lowl (RRID:IMSR_JAX:016963), Slc32a1tm2(cre)Lowl (RRID:IMSR_JAX:016962), Slc6a3tm1.1(cre)Bkmn (RRID:IMSR_JAX:006660) and Slc32a1tm1.1(flpo)Hze (RRID:IMSR_JAX:029591). The DAT-FlpO (Slc6a3em1(flpo)Hbat; RRID:IMSR_JAX:035436) mice were generously provided by Dr. Helen Bateup (UC Berkeley). Animals were on average 15-weeks old at the time of surgery and 28-weeks old by the end of the experiments. Mice were group-housed and maintained on a 12h light-dark cycle (i.e. light cycle; 7am-7pm) with food and water available ad libitum unless noted. All experiments were conducted during the light phase of the cycle. Housing and procedure rooms were maintained at a temperature of ~21C and humidity of ~50%.

Wild animals

No wild animals were used in this study.

Reporting on sex

Both males and females were used in all experiments. Data are disaggregated by sex in Table S2 and in Source Data files.

Field-collected samples

No field collected samples were used in the study.

Ethics oversight

All protocols were approved by the University of California San Diego Institutional Animal Care and Use Committee

Note that full information on the approval of the study protocol must also be provided in the manuscript.
